# *YIPF5* mutations cause neonatal diabetes and microcephaly through endoplasmic reticulum stress

**DOI:** 10.1172/JCI141455

**Published:** 2020-11-09

**Authors:** Elisa De Franco, Maria Lytrivi, Hazem Ibrahim, Hossam Montaser, Matthew N. Wakeling, Federica Fantuzzi, Kashyap Patel, Céline Demarez, Ying Cai, Mariana Igoillo-Esteve, Cristina Cosentino, Väinö Lithovius, Helena Vihinen, Eija Jokitalo, Thomas W. Laver, Matthew B. Johnson, Toshiaki Sawatani, Hadis Shakeri, Nathalie Pachera, Belma Haliloglu, Mehmet Nuri Ozbek, Edip Unal, Ruken Yıldırım, Tushar Godbole, Melek Yildiz, Banu Aydin, Angeline Bilheu, Ikuo Suzuki, Sarah E. Flanagan, Pierre Vanderhaeghen, Valérie Senée, Cécile Julier, Piero Marchetti, Decio L. Eizirik, Sian Ellard, Jonna Saarimäki-Vire, Timo Otonkoski, Miriam Cnop, Andrew T. Hattersley

**Affiliations:** 1 Institute of Biomedical and Clinical Science, University of Exeter Medical School, Exeter, United Kingdom.; 2 ULB Center for Diabetes Research and; 3 Division of Endocrinology, Erasmus Hospital, Université Libre de Bruxelles, Brussels, Belgium.; 4 Stem Cells and Metabolism Research Program, Faculty of Medicine, University of Helsinki, Helsinki, Finland.; 5 Endocrinology and Metabolism, Department of Medicine and Surgery, University of Parma, Parma, Italy.; 6 Electron Microscopy Unit, Institute of Biotechnology, University of Helsinki, Helsinki, Finland.; 7 Yeditepe University Hospital, Istanbul, Turkey.; 8 Gazi Yaşargil Education and Research Hospital, Diyarbakır, Turkey.; 9 Dicle University, Faculty of Medicine, Department of Pediatric Endocrinology, Diyarbakır, Turkey.; 10 Harmony Health Hub, Nashik, India.; 11 Istanbul University, Istanbul Faculty of Medicine, Department of Pediatric Endocrinology, Istanbul, Turkey.; 12 Kanuni Sultan Suleyman Training and Research Hospital, Department of Pediatric Endocrinology, Istanbul, Turkey.; 13 Institute of Interdisciplinary Research (IRIBHM), ULB Neuroscience Institute, Université Libre de Bruxelles, Brussels, Belgium.; 14 VIB-KU Leuven Center for Brain & Disease Research, Leuven, Belgium.; 15 Department of Neurosciences, Leuven Brain Institute, KU Leuven, Leuven, Belgium.; 16 Welbio, Université Libre de Bruxelles, Brussels, Belgium.; 17 Université de Paris, Faculté de Médecine Paris–Diderot, U958, Paris, France.; 18 Department of Clinical and Experimental Medicine, University of Pisa, Pisa, Italy.; 19 Indiana Biosciences Research Institute, Indianapolis, Indiana, USA.; 20 Children’s Hospital, University of Helsinki and Helsinki University Hospital, Helsinki, Finland.

**Keywords:** Cell Biology, Genetics, Cell stress, Diabetes, Human stem cells

## Abstract

Neonatal diabetes is caused by single gene mutations reducing pancreatic β cell number or impairing β cell function. Understanding the genetic basis of rare diabetes subtypes highlights fundamental biological processes in β cells. We identified 6 patients from 5 families with homozygous mutations in the *YIPF5* gene, which is involved in trafficking between the endoplasmic reticulum (ER) and the Golgi. All patients had neonatal/early-onset diabetes, severe microcephaly, and epilepsy. *YIPF5* is expressed during human brain development, in adult brain and pancreatic islets. We used 3 human β cell models (*YIPF5* silencing in EndoC-βH1 cells, *YIPF5* knockout and mutation knockin in embryonic stem cells, and patient-derived induced pluripotent stem cells) to investigate the mechanism through which *YIPF5* loss of function affects β cells. Loss of *YIPF5* function in stem cell–derived islet cells resulted in proinsulin retention in the ER, marked ER stress, and β cell failure. Partial *YIPF5* silencing in EndoC-βH1 cells and a patient mutation in stem cells increased the β cell sensitivity to ER stress–induced apoptosis. We report recessive *YIPF5* mutations as the genetic cause of a congenital syndrome of microcephaly, epilepsy, and neonatal/early-onset diabetes, highlighting a critical role of *YIPF5* in β cells and neurons. We believe this is the first report of mutations disrupting the ER-to-Golgi trafficking, resulting in diabetes.

## Graphical abstract

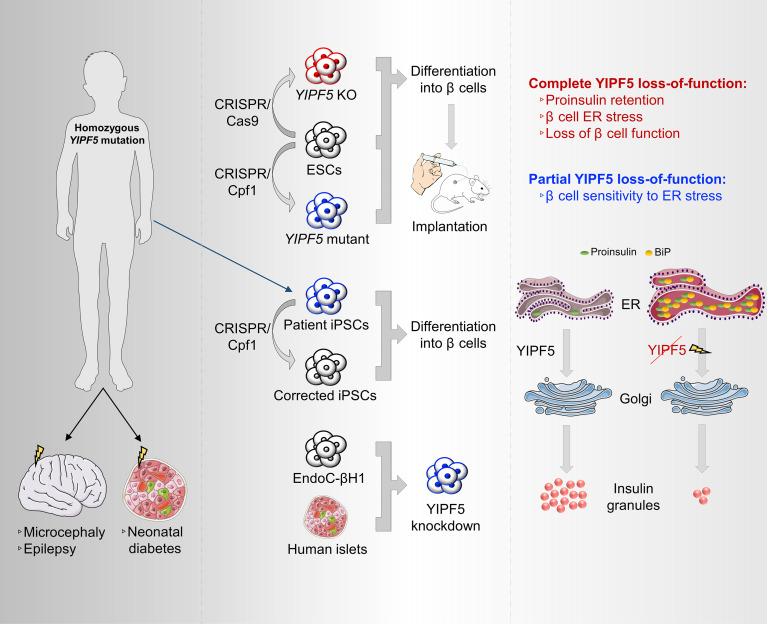

## Introduction

Neonatal diabetes mellitus develops before 6 months of age and is caused by reduced pancreatic β cell number (reduced formation/increased destruction) or impaired β cell function. Previous studies have shown that neonatal diabetes is most likely caused by a mutation in a single gene, rather than being autoimmune type 1 diabetes (1, 2). To date, 30 genetic causes have been described, which account for 82% of cases (3–9). Additional clinical features are often present in patients with neonatal diabetes, with 18% of them having neurological symptoms (3). This is not surprising, as β cells and neurons have key genes and cellular functions in common (10, 11).

Pathogenic variants in 11 genes (*ABCC8*, *KCNJ11*, *CNOT1*, *EIF2AK3*, *SLC19A2*, *IER3IP1*, *PTF1A*, *NEUROD1*, *MNX1*, *WFS1*, and *NKX2-2*) are known to cause neonatal diabetes with neurological features, ranging from developmental delay to structural abnormalities such as microcephaly (3, 4). Recently, pathogenic variants in 3 genes (*TRMT10A* [ref. 12], *PPP1R15B* [ref. 13], and *EIF2S3* [refs. 14, 15]) were reported to cause young- or adult-onset diabetes and microcephaly. The overlap between genes causing diabetes and neurological features highlights shared pathways that are critically important for development (*CNOT1*, *PTF1A*, *NEUROD1*, *MNX1*, and *NKX2-2*) and function (*ABCC8*, *KCNJ11*, *EIF2AK3*, *SLC19A2*, *IER3IP1*, *WFS1*, *TRMT10A*, *PPP1R15B*, *EIF2S3*) of both β cells and neurons.

One of the pathways known to be crucial for the function of both β and brain cells is the endoplasmic reticulum (ER) stress response. Pathogenic variants in 8 genes known to be involved in regulating the ER stress response have been found to cause diabetes (ranging from neonatal to adolescent/adult-onset diabetes), often associated with neurological features (6, 16). The ER stress response is an adaptive pathway that is triggered when there is an imbalance in the ability of the ER to fold proteins and the cellular protein folding demand, leading to accumulation of unfolded or misfolded proteins in the ER. This can happen when a genetic mutation results in a wrongly folded protein that is unable to exit the ER, as happens with dominant *INS* mutations causing diabetes (17). ER stress can also be triggered when transport of folded proteins from the ER to the Golgi compartment is compromised (18, 19). The ER stress response aims at slowing down translation of new proteins (through PERK-mediated eIF2α phosphorylation), while increasing the ER’s protein folding ability. Mutations in 5 genes that dysregulate signaling in the PERK branch of the ER stress response cause β cell dysfunction and apoptosis by perturbing translational control.

Identifying the genes causing syndromic forms of neonatal diabetes that include neurological features can highlight pathways important for development and function of β cells and neurons, giving insights into the pathogenesis of more common diseases. In this study, we used genome sequencing to identify recessive pathogenic variants in *YIPF5* as the genetic cause of a congenital syndrome characterized by neonatal/early-onset diabetes, severe microcephaly, and epilepsy. Functional studies in human β cell models highlight the importance of ER-to-Golgi trafficking in β cells and neurons.

## Results

### Genetic analysis.

**Figure 1:**
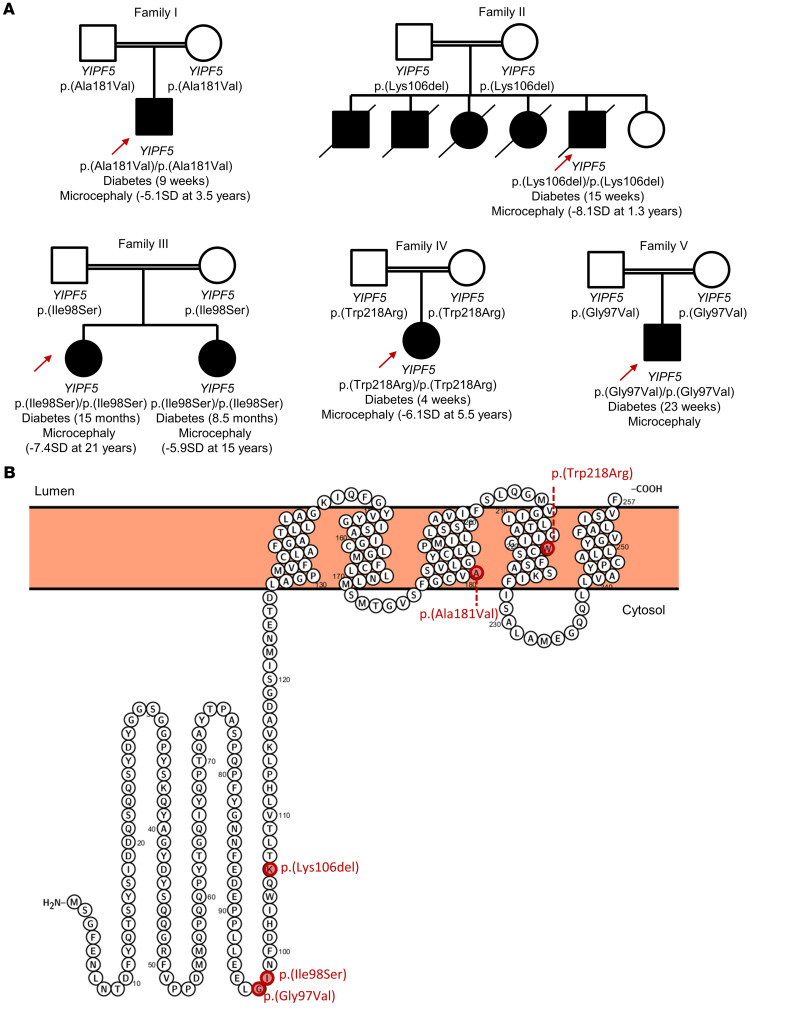
Identification of homozygous *YIPF5* mutations in 6 patients with neonatal diabetes, severe microcephaly, and epilepsy. (**A**) Partial pedigrees and summary of clinical features of the 6 patients with homozygous *YIPF5* mutations. Age at diagnosis of diabetes and head circumference standard deviation below the mean are given in parentheses. (**B**) Schematic representation of the YIPF5 ER transmembrane protein using the CCTOP in silico predictor (http://cctop.enzim.ttk.mta.hu/). Note that there is uncertainty regarding YIPF5 transmembrane predictions and the position of the p.Trp218 residue is predicted to be cytoplasmic by UniProtKB (https://www.uniprot.org/).

Genome sequencing was performed for 2 unrelated probands diagnosed with neonatal diabetes, epilepsy, and severe microcephaly (patients I and II in Table 1 and Figure 1A) in whom mutations in known neonatal diabetes genes had been excluded. Since both patients were born to consanguineous (first cousins) parents and the phenotype was strikingly similar, we hypothesized that both were affected by the same autosomal recessive condition. We therefore focused our analysis on homozygous rare coding variants in shared genes.

Rare homozygous coding variants in 28 genes were identified in patient I, who had genome-wide homozygosity of 11.7% calculated from genome sequencing data. Patient II had genome-wide homozygosity of 6.9%, and rare homozygous coding variants were identified in 10 genes (see Supplemental Tables 1 and 2 and Supplemental Figure 1A; supplemental material available online with this article; https://doi.org/10.1172/JCI141455DS1). The only gene in common in both individuals was *YIPF5*, with patient I being homozygous for a missense, p.(Ala181Val), and patient II harboring a homozygous in-frame deletion variant, p.(Lys106del).

Neither variant was listed in the gnomAD database (>120,000 individuals [ref. 20], accessed May 18, 2020), and both affect residues that are highly conserved across species (up to *Saccharomyces cerevisiae*). The p.(Ala181Val) amino acid change was predicted to be likely to affect protein function by 2 of 4 tools used to assess the effect of missense variants, Sorting Intolerant from Tolerant (SIFT) (21) and MutationTaster (22) (Supplemental Table 3). Testing for the mutations in parental samples confirmed that the unaffected parents were heterozygous for the mutations.

To replicate this finding in a larger unselected cohort, we analyzed the coding regions and exon-intron boundaries of the *YIPF5* gene in a further 187 cases diagnosed with diabetes before the age of 12 months (10 also had microcephaly) who did not have a mutation in known monogenic diabetes genes. We identified 3 homozygous *YIPF5* missense mutations, p.(Ile98Ser), p.(Trp218Arg), and p.(Gly97Val), in 3 cases. Testing for the mutations in family members confirmed that the parents were all heterozygous for the mutations and that patient III’s affected sister was also homozygous for the p.(Ile98Ser) variant (Figure 1A). The 3 variants are not listed in gnomAD, affect residues that are conserved though species up to *S*. *cerevisiae*, and are predicted by 4 of 4 in silico tools — Align GVGD (23), SIFT, PolyPhen-2 (24), and MutationTaster — to be likely to affect YIPF5 protein (Supplemental Table 3).

Protein domain analysis using CCTOP (25) predicted 3 of the variants, the p.(Gly97Val), p.(Ile98Ser), and p.(Lys106del), to affect residues located in the cytoplasmic domain, while the p.(Ala181Val) and p.(Trp218Arg) variants affect residues located in the third and fourth transmembrane domain, respectively (Figure 1B). The nature and position of the variants are consistent with at least a partial loss of protein function.

### Clinical evaluation.

The clinical features of the 6 patients are summarized in Table 1. All had severe microcephaly (median standard deviation score –6.2, IQR –6.5 to 6.1; Supplemental Figure 1B), epilepsy diagnosed in the neonatal period (range 1–7 months), and neonatal/early-onset diabetes (age at diagnosis range 4 weeks to 20 months) that was treated with a full replacement dose of insulin. For all 6 patients, the birth weight was low (median standard deviation score –1.85 (–1.99 to –1.72)), consistent with reduced insulin secretion in utero.

Patient II died at the age of 1.3 years. There was a family history of 4 siblings (2 female and 2 male) diagnosed with neonatal diabetes, epilepsy, and microcephaly who had died in infancy. DNA was not available for these individuals.

Five individuals (II, IIIa, IIIb, IV, and V) were reported to have severe developmental delay, while neuromotor development was reported to be normal in patient I, who was 5 years of age at time of writing. No other clinical features were reported.

There was no significant family history of diabetes for any of the patients. Patient IV’s father, who was diagnosed with type 2 diabetes and was being treated with oral hypoglycemic agents, was the only one of the patients’ parents to be affected with diabetes.

### YIPF5 is expressed in human islets and brain.

**Figure 2:**
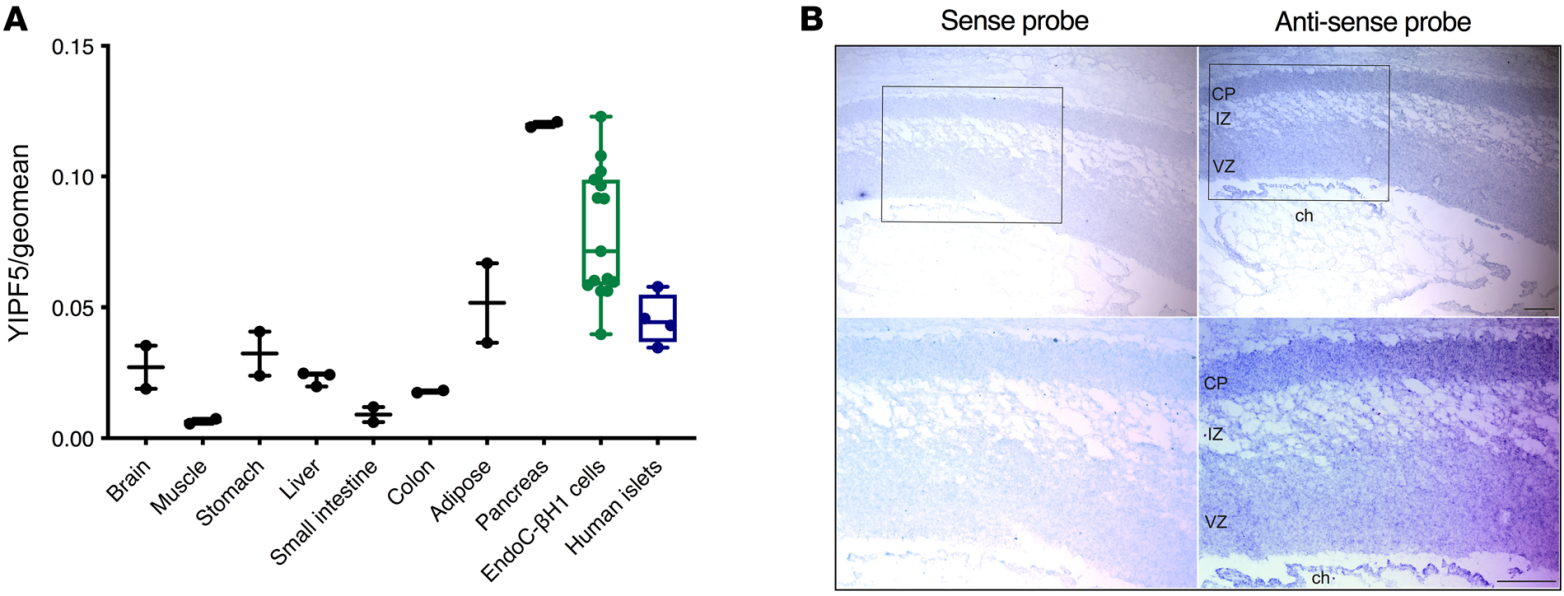
YIPF5 is expressed in human pancreatic tissue and brain. (**A**) YIPF5 mRNA expression was measured by qPCR in human tissues (*n* = 2–3), EndoC-βH1 cells (*n* = 15), and human islets (*n* = 4) and normalized to the geometric mean of the reference genes ACTB, GAPDH, and OAZ1. (**B**) In situ hybridization of YIPF5 in human fetal cortex at gestational week 12. Expression is found in the ventricular zone (VZ), intermediate zone (IZ), and cortical plate (CP) as well choroid plexus (ch) (antisense probe, right). No signal was detected when the sense probe was used (negative control, left). Scale bar: 100 μm.

*YIPF5* mRNA expression was evaluated in human tissues by quantitative (qPCR). *YIPF5* was ubiquitously expressed, with abundant expression in pancreatic tissue, islets, β cells, and brain (Figure 2A).

The expression pattern of *YIPF5* during brain development was examined by in situ hybridization in human fetal brain samples encompassing stages 12 to 21 gestational weeks (Figure 2B and data not shown). This revealed significant broad expression of *YIPF5* in the developing cortex at all stages examined but most strikingly at 12 gestational weeks. Expression was found in both progenitor (ventricular zone) and neuronal (intermediate zone and cortical plate) compartments. Some selective expression could also be detected within the choroid plexus within the cerebral ventricles. No significant signal was observed with sense probes, confirming the specificity of the findings (Figure 2B).

### YIPF5 deficiency sensitizes human β cells to ER stress–induced apoptosis.

**Figure 3:**
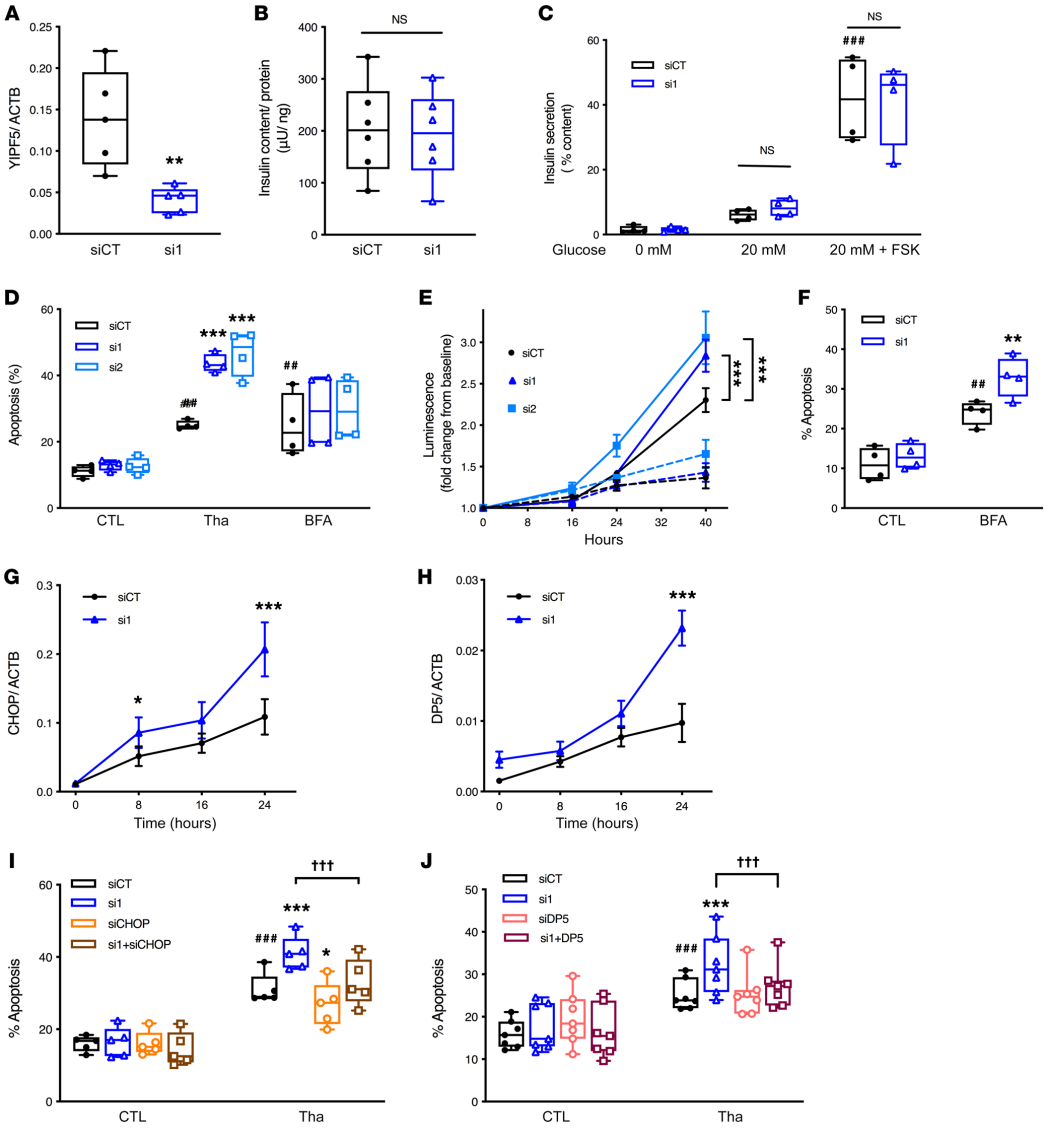
YIPF5 deficiency does not affect insulin secretion but sensitizes β cells to ER stress–induced apoptosis. (**A**–**C**) EndoC-βH1 cells were transfected with siRNA against *YIPF5* (si1) or control siRNA (siCT) for 48 hours and incubated with 0 or 20 mM glucose or 20 mM glucose plus 10 μM forskolin (FSK). (**A**) YIPF5 mRNA expression by qPCR. (**B**) Insulin content normalized for total protein content. (**C**) Insulin secretion expressed as percentage of total insulin content. (**D** and **E**) EndoC-βH1 cells were transfected with 2 siRNAs against YIPF5 (si1 and si2) or control siRNA (siCT) for 48 hours and exposed or not (CTL) to thapsigargin (Tha) for 40 hours or brefeldin A (BFA) for 16 hours (*n* = 4). Apoptosis was evaluated by staining with DNA-binding dyes (*n* = 4) (**D**) or luminescence produced by annexin V binding (RealTime-Glo Annexin V assay) at the indicated time points (*n* = 3) (**E**). Thapsigargin is presented by solid lines and nontreated cells by dashed lines. (**F**) Dispersed human islet cells were transfected with si1 or siCT for 48 hours and exposed or not to brefeldin A for 24 hours. Apoptosis was evaluated by staining with DNA-binding dyes (*n* = 4). (**G** and **H**) EndoC-βH1 cells were transfected with si1 or siCT for 48 hours and exposed to thapsigargin for the indicated times (*n* = 5–6). CHOP (**G**) and DP5 (**H**) mRNA expression was measured by qPCR, normalized to β-actin (ACTB). (**I** and **J**) EndoC-βH1 cells were transfected with siCT or si1 and/or siRNA against CHOP (siCHOP) (**I**) or DP5 (siDP5) (**J**) and treated or not with thapsigargin for 40 hours (*n* = 5 and *n* = 8, respectively). Apoptosis was examined by DNA-binding dye. Individual symbols represent independent experiments, and box plots show the median by a horizontal line, 25th and 75th percentiles at the bottom and top of the boxes, and minimum and maximum values by whiskers. In time course experiments, data are shown as mean ± SEM. Paired 2-way ANOVA or mixed-model analysis (in case of missing values) followed by Bonferroni post hoc test. *P < 0.05, **P < 0.01, ***P < 0.001 vs. siCT in respective condition; ^##^*P* < 0.01, ^###^*P* < 0.001 for treated vs. untreated cells; ^†††^*P* < 0.001 as indicated.

To investigate the effect of YIPF5 loss in β cells, we established an in vitro model of YIPF5 deficiency using RNA interference in human EndoC-βH1 cells. *YIPF5* was efficiently silenced using 2 different siRNAs by 50%–75% at the mRNA level (Figure 3A and Supplemental Figure 2) and by approximately 50% at the protein level (*n* = 2–4).

YIPF5 depletion did not impact β cell function: glucose- and forskolin-stimulated insulin secretion was comparable in YIPF5-depleted and -competent EndoC-βH1 cells, as was insulin content (Figure 3, B and C). Furthermore, YIPF5 depletion did not affect proliferation rates of EndoC-βH1 cells, assessed by Ki67 immunostaining (data not shown).

Survival of β cells was evaluated under basal condition and following exposure to the ER stressors brefeldin A (which blocks ER-to-Golgi transport) and thapsigargin (which inhibits the sarco/endoplasmic reticulum Ca^2+^ ATPase [SERCA]). *YIPF5* knockdown did not significantly affect basal β cell survival, but YIPF5-depleted β cells were markedly sensitized to thapsigargin (Figure 3D). This was confirmed by a second apoptosis assay that measures annexin V binding in real time, showing that thapsigargin induced more apoptosis in cells transfected with either *YIPF5* siRNA (Figure 3E). Brefeldin A treatment markedly induced *YIPF5* mRNA expression in EndoC-βH1 cells and human islets (Supplemental Figure 2, A and B); a trend for YIPF5 protein induction was seen in EndoC-βH1 cells (approximately 2-fold; *n* = 4). *YIPF5* silencing enhanced apoptosis in brefeldin-treated clonal β cells and human islets (Figure 3, D and F), in keeping with the presumed function of YIPF5 in ER-to-Golgi trafficking.

### YIPF5 deficiency increases human β cell ER stress signaling and induces proapoptotic proteins PUMA and DP5.

We next investigated whether YIPF5 deficiency affects ER stress signaling by measuring mRNA expression of *CHOP*, spliced *XBP1* (sXBP1), *BiP*, *PDIA4*, and *HYOU1*, which act in the 3 canonical branches of the ER stress response (downstream of PERK, IRE1, and ATF6, respectively). Time course experiments in EndoC-βH1 cells exposed to thapsigargin showed that *YIPF5* knockdown induced ER stress markers (Figure 3G and Supplemental Figure 2, C–F). The induction was more pronounced for PERK- and ATF6-dependent markers, while the IRE1 target sXBP1 was induced to a lesser extent. In brefeldin-treated cells, *YIPF5* silencing also enhanced ER stress signaling (data not shown). Taken together, these results show that YIPF5 deficiency potentiates the ER stress response.

The BH3-only proteins PUMA (also known as BBC3), DP5 (also known as HRK), and BIM (also known as BCL2L11) activate apoptosis downstream of ER stress, playing a central role in β cell demise (26–29). In time course experiments, *DP5* expression was induced by *YIPF5* silencing in thapsigargin-exposed (Figure 3H) and brefeldin-exposed cells (data not shown). *PUMA* expression was also induced by *YIPF5* silencing (Supplemental Figure 2G), while *BIM* expression was not altered.

In order to examine whether the induction of CHOP, a proapoptotic transcription factor in the PERK branch of the ER stress response, sensitizes YIPF5-deficient β cells to apoptosis, we double-knocked-down *CHOP* and *YIPF5* (Figure 3I and Supplemental Figure 2H). *CHOP* silencing protected YIPF5-depleted cells from thapsigargin (Figure 3I), and similarly, *DP5* and *YIPF5* double knockdown (Supplemental Figure 2I) partially protected β cells from thapsigargin-induced apoptosis (Figure 3J).

### Proinsulin accumulation, increased ER stress signaling, and reduced insulin content in YIPF5-knockout stem cell–derived β cells.

**Figure 4:**
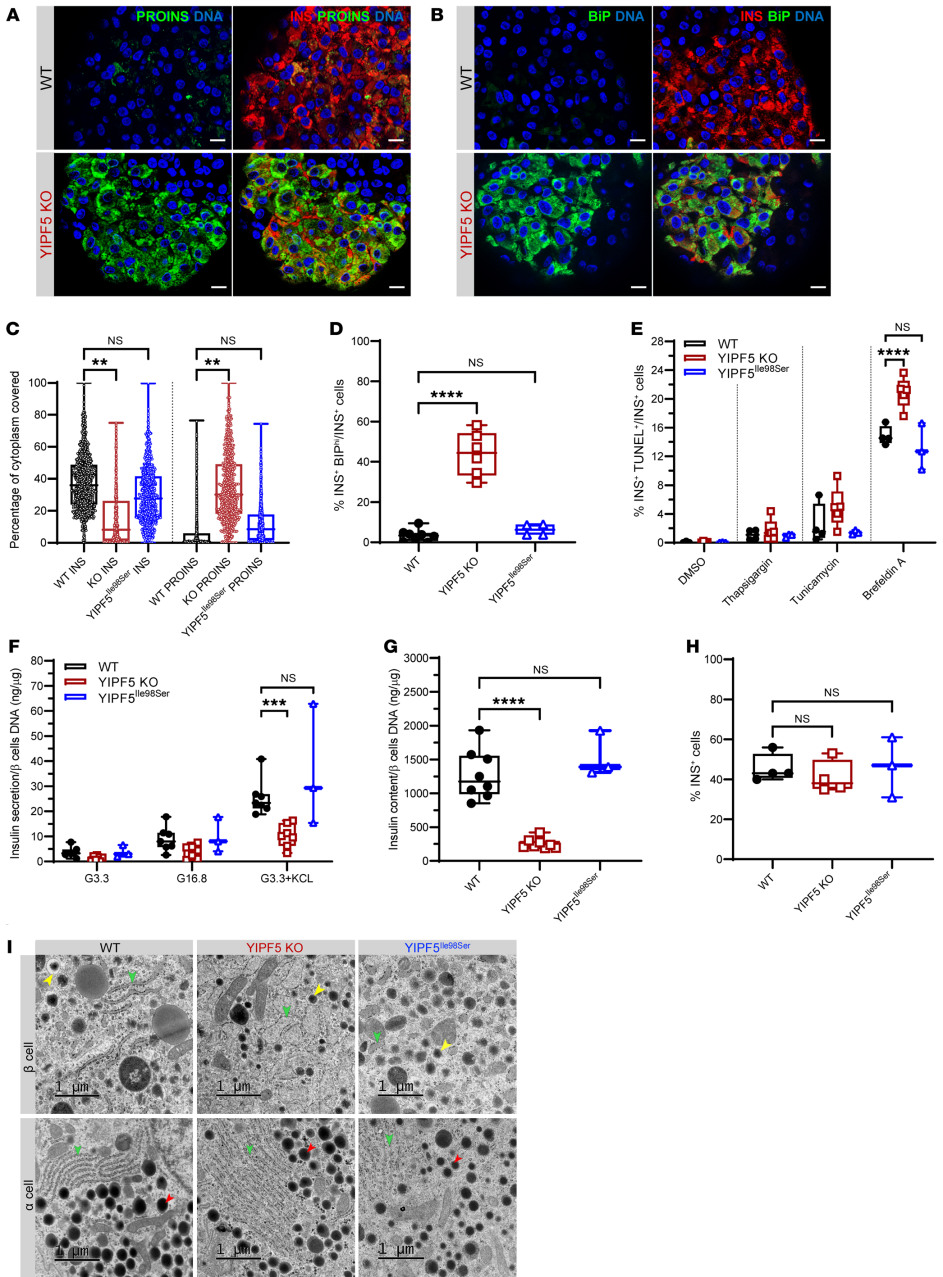
Proinsulin accumulation, increased ER stress signaling, and reduced insulin content in YIPF5-knockout stem cell–derived β cells. (**A**) Immunocytochemistry for proinsulin (PROINS) and insulin (INS) at stage 7 of in vitro differentiation for WT and YIPF5-KO cells. Scale bars: 25 μm. (**B**) Immunocytochemistry for BiP and insulin (INS) at stage 7 of in vitro differentiation. Scale bars: 25 μm. (**C**) Percentage of cytoplasmic area covered by proinsulin or insulin per insulin-positive cell (*n* = 3). (**D**) Percentage of INS^+^BiP^hi^ cells per total number of INS^+^ cells (*n* = 4–8). (**E**) Percentage of apoptotic cells (INS^+^TUNEL^+^) per total number of INS^+^ cells after treatment with vehicle (DMSO) and the ER stressors thapsigargin, tunicamycin, and brefeldin A (*n* = 3–5). (**F**) Static glucose-stimulated insulin secretion at stage 7 normalized to micrograms DNA of β cells (*n* = 3–7). (**G**) Insulin content of stage 7 differentiated cells normalized to micrograms DNA of β cells (*n* = 3–8). (**H**) Percentage of INS^+^ cells at week 2 of stage 7 (*n* = 3–4). Statistical significance was assessed in **C**, **D**, **G**, and **H** by 1-way ANOVA test with Bonferroni correction, and in **E** and **F** by 2-way ANOVA test with Bonferroni correction. **P < 0.01, ***P < 0.001, ****P < 0.0001. Error bars represent SD from the mean. (**I**) Transmission electron microscopy of WT, YIPF5-KO, and YIPF5^Ile98Ser^ stage 7 cells showing the cytoplasmic area of β and α cells. Yellow arrowheads point at insulin granules, red arrowheads at glucagon granules, and green arrowheads at ER. Scale bars: 1 μm.

To study the role of YIPF5 in the development and function of pancreatic β cells, we used CRISPR/Cas9 technology to generate a *YIPF5* knockout (KO) in the human embryonic stem cell (hESC) line H1. We deleted exon 3, which is common to all the *YIPF5* isoforms (Supplemental Figure 3, A and B). The *YIPF5*-KO cell line expressed pluripotency markers as expected and showed a normal karyotype (Supplemental Figure 4, A–C) with no evidence of CRISPR-induced off-target indels (data not shown). In addition to the KO, we generated an isogenic YIPF5^Ile98Ser^ mutation using CRISPR/Cpf1–mediated homology-directed repair (HDR) (Supplemental Figure 5). This is the same mutation present in the 2 siblings in family III, who were both diagnosed with diabetes after the age of 6 months. The H1 wild-type (WT), KO, and YIPF5^Ile98Ser^ cells differentiated normally until the pancreatic endocrine stage. At this stage, proinsulin accumulation was evident in the KO β cells (Figure 4A and Supplemental Figure 6A). The percentage of cytoplasmic area stained for proinsulin per β cell of the KO was 5.5-fold higher than in their WT counterparts, while the percentage of insulin area was 70% less in the KO β cells (Figure 4C). The YIPF5^Ile98Ser^ β cells showed a much milder phenotype with 20% less insulin area and a 1.7-fold increase in proinsulin area compared with the WT; however, the differences were not statistically significant. A 15-fold increase was detected in the number of cells with high BiP immunoreactivity (INS^+^BiP^hi^) in the KO (Figure 4, B and D; and Supplemental Figure 6B), likely reflecting ER stress response triggered by proinsulin retention in the ER. Consistent with this, the KO cells showed significant induction of *BiP* and *HYOU1* mRNA expression at stage 7 of differentiation, but not of *ATF6*, *XBP1s*, and *CHOP* (Supplemental Figure 7). *INS* mRNA expression was significantly reduced at stages 6 and 7 in the KO (Supplemental Figure 7), although there was no difference in the percentage of INS^+^ cells in the 3 cell lines (Figure 4H). In the KO β cells, no increase in apoptosis was detected by TUNEL assay at stage 7, but the cells were more sensitive following exposure to chemical ER stressors, especially brefeldin A (Figure 4E). Stem cell–derived β cells of all 3 lines showed glucose-stimulated insulin secretion, but the absolute amount of secreted insulin was 50% lower in the KO cells (Figure 4F), and cellular insulin content was reduced by 80% (Figure 4G). Transmission electron microscopy was used to study the ultrastructure of the stem cell–derived endocrine cells. The ER morphology identified by the studded ribosomes along its outer membrane showed a marked distension of the ER cisternae in all of the studied KO β cells, while the ER in α cells was not affected. ER dilation was observed in only a minority of the YIPF5^Ile98Ser^ β cells (Figure 4I and Supplemental Figure 8).

### Loss of β cell function after in vivo implantation.

**Figure 5:**
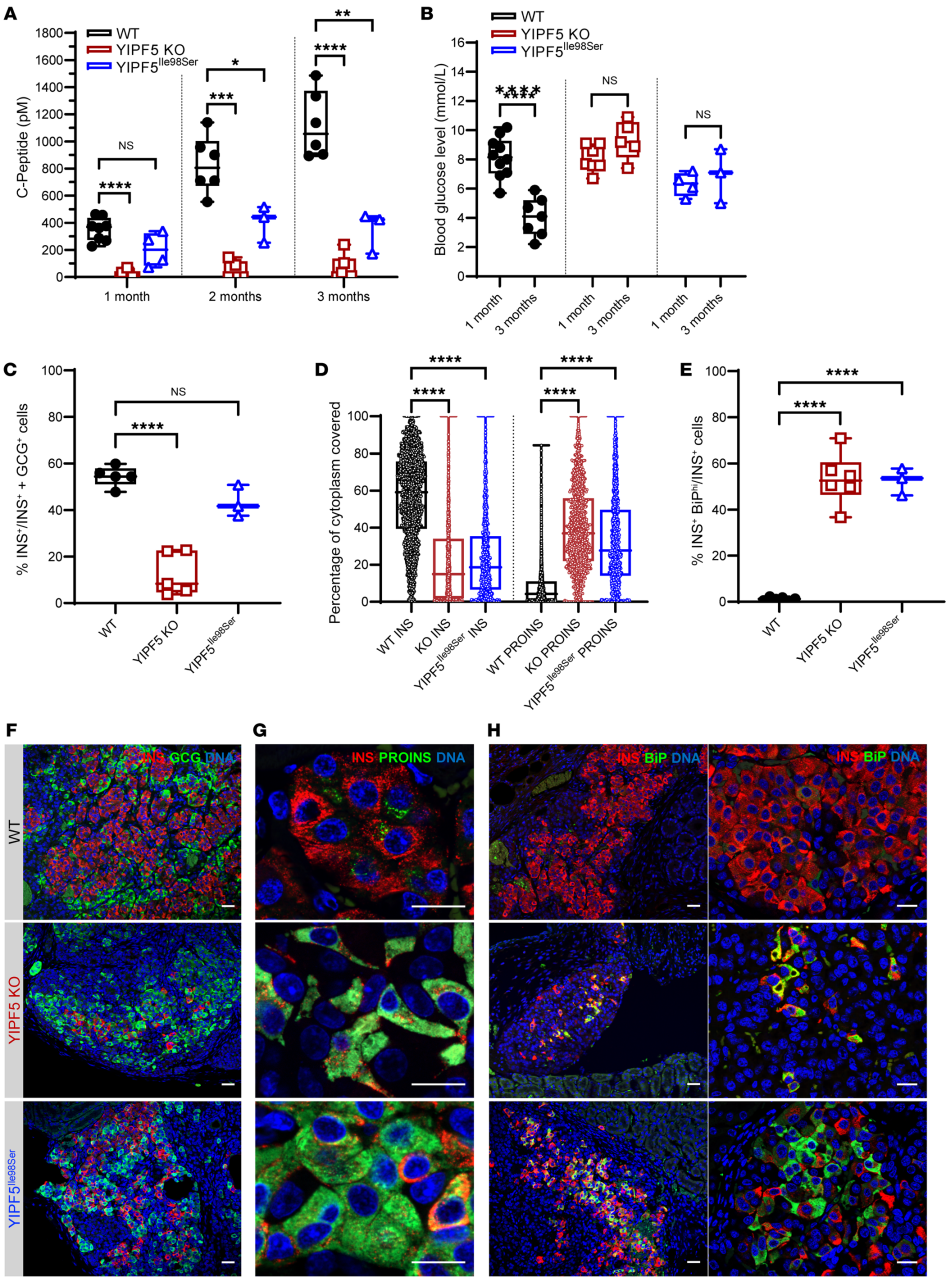
Reduced C-peptide secretion and β cell numbers in implanted YIPF5 knockout and signs of YIPF5^Ile98Ser^ β cell failure. (**A**) Human C-peptide levels measured in mouse serum through 3 months after implantation (*n* = 3–8). (**B**) Mouse blood glucose levels at 1 and 3 months after implantation (*n* = 3–10). (**C**) Percentage of INS^+^GCG^–^ cells per the total number of INS^+^ plus GCG^+^ cells (*n* = 3–5). (**D**) Percentage of cytoplasmic area covered by proinsulin or insulin in insulin-positive cells (*n* = 3–4). (**E**) Percentage of INS^+^BiP^hi^ cells per total number of INS^+^ cells (*n* = 3–6). (**F**–**H**) Immunohistochemistry of grafts for glucagon (GCG) and insulin (INS) (**F**), proinsulin (PROINS) and insulin (INS) (**G**), and BiP and insulin (INS) (**H**) 3 months after implantation. Scale bars: 100 μm (**F**); 25 μm (**G**); 100 μm (**H**, left); 25 μm (**H**, right). Statistical significance was assessed by 2-way ANOVA test with Bonferroni correction in **A**, by multiple *t* test with Bonferroni correction in **B**, and by 1-way ANOVA test with Bonferroni correction in **C**–**E**. *P < 0.05, **P < 0.01, ***P < 0.001, ****P < 0.00001. Error bars represent SD from the mean.

Stage 7 islet-like aggregates differentiated from WT, KO, and YIPF5^Ile98Ser^ H1 stem cells were implanted under the kidney capsule of immunocompromised NOD/SCID-γ mice, and their function was monitored by measurement of the serum levels of human C-peptide. While the WT-implanted mice reached a level of 1 nM at 3 months, human C-peptide was barely detectable in the KO-implanted mice, consistent with impaired β cell function. Lower C-peptide levels were also recorded in the YIPF5^Ile98Ser^-implanted mice after 1, 2, and 3 months (Figure 5A). Blood glucose levels in the WT-implanted mice dropped from 8 to 4 mM 3 months after implantation, reflecting glycemic regulation by transplanted human β cells, while no such effect was observed in the KO- or YIPF5^Ile98Ser^-implanted mice (Figure 5B).

The grafts were retrieved for immunohistochemical analysis 3 months after implantation. Insulin- and glucagon-positive cells, which were the dominant cell types, were quantified. In the WT and YIPF5^Ile98Ser^ grafts, 55% and 42% of the cells were insulin-positive, respectively. In contrast, glucagon-positive cells dominated in the KO grafts, where only 12% of the grafted cells were insulin-positive (Figure 5, C and F). The mature β cells in the KO and YIPF5^Ile98Ser^ grafts showed a 3.3- and 3-fold reduction, respectively, in the percentage of cytoplasmic insulin-positive area, and a 5.7- and 5.4-fold increase, respectively, in the proinsulin area (Figure 5, D and G). The accumulated proinsulin colocalized with the ER proteins calreticulin, BiP, and GRP170 (Supplemental Figure 9). The insulin-positive KO cells sustained high BiP expression in vivo, consistent with persistent ER stress. Grafts of YIPF5^Ile98Ser^-mutant aggregates also showed clear signs of increased ER stress when harvested at 3 months after implantation (Figure 5, E and H). No difference was detected in the number of TUNEL^+^ cells in the grafts (data not shown).

*β Cells from patients’ induced pluripotent stem cellsharboring the p.(Ile98Ser) mutation are sensitive to ER stress–induced apoptosis*. To further assess the impact of one of the *YIPF5* missense mutations in a directly patient-relevant model, we generated patients’ induced pluripotent stem cells (iPSCs) and differentiated them into pancreatic endocrine cells. PBMCs were obtained from patients IIIa and IIIb, who are both homozygous for the p.(Ile98Ser) mutation, and reprogrammed into iPSCs using Sendai virus. The 4 iPSC lines (2 from each patient) had normal karyotype, expressed pluripotency markers, lost the expression of the exogenous transgene vector, and successfully differentiated into the 3 germ layers in an embryoid body assay (Supplemental Figure 10, A–E). For one patient iPSC line, we corrected the mutation by CRISPR/Cpf1 and generated 2 isogenic control iPSC lines (Supplemental Figure 5A). These corrected iPSCs had normal morphology and karyotype and expressed pluripotency markers (Supplemental Figure 5, D–F).

**Figure 6:**
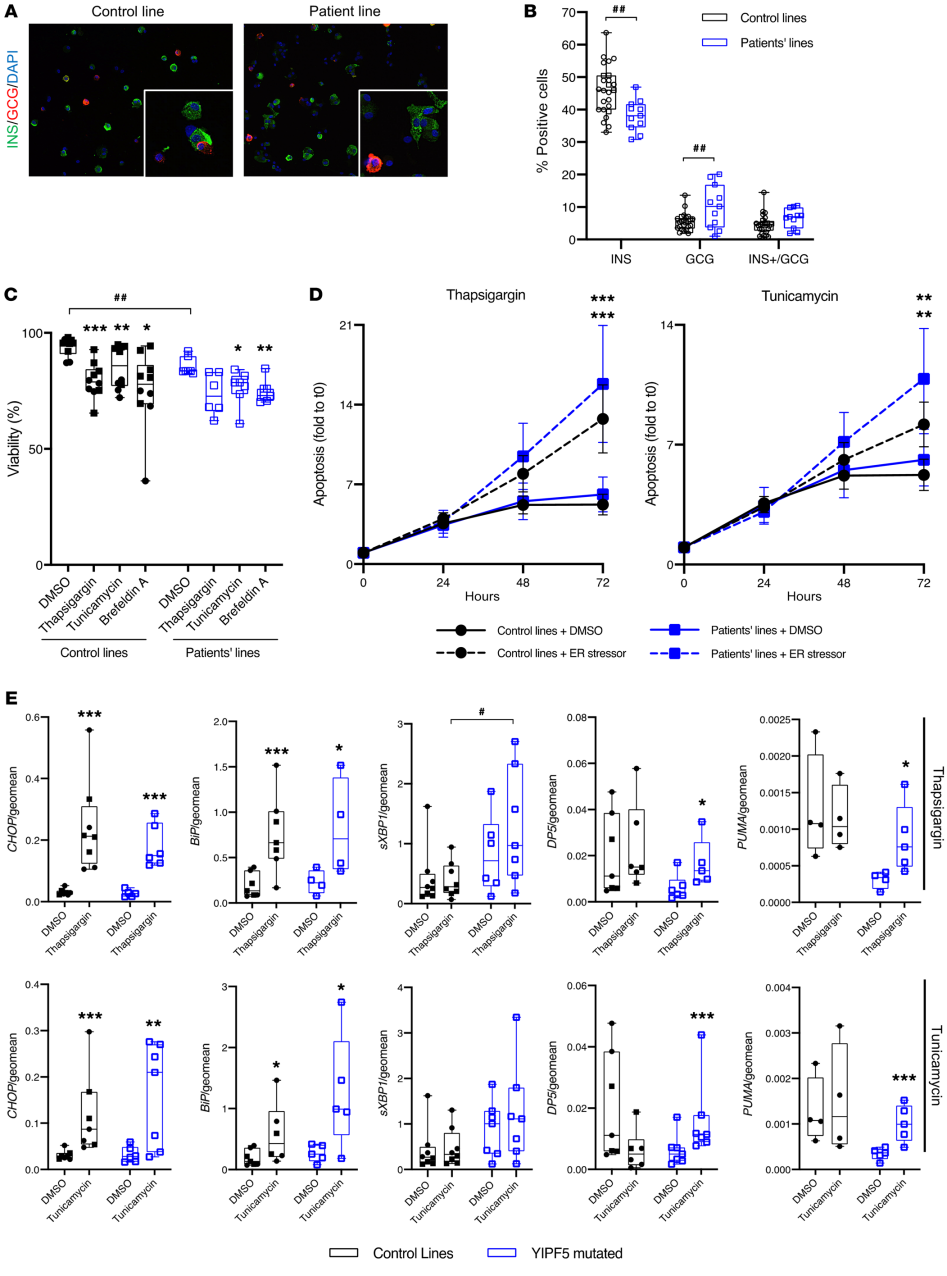
iPSCs from patients IIIa and IIIb differentiated into β cells are sensitive to ER stress–induced apoptosis. (**A**) Representative immunostaining of dispersed stage 7 aggregates stained for insulin (INS, green) and glucagon (GCG, red). Nuclei were visualized with DAPI (blue). (**B**) Quantification of immunostained cells (expressed as percent of total cells) in dispersed stage 7 control (*n* = 25) and patient cells (*n* = 11). Blue squares represent patient cells (2 patients, 2 iPSC lines for each); black circles and squares represent healthy control (1 iPSC line) and corrected patient cells (2 iPSC lines from 1 patient), respectively. (**C** and **D**) Apoptosis was assessed by staining with DNA-binding dyes in vehicle- (DMSO-)treated, thapsigargin-treated, and tunicamycin-treated control and corrected (*n* = 10) and patient (*n* = 6–7) stage 7 aggregates (**C**) or by luminescence produced by annexin V binding in time course experiments (means ± SEM; *n* = 10 control and corrected lines and *n* = 5 patient lines) (**D**). (**E**) mRNA expression of CHOP, BiP, sXBP1, DP5, and PUMA assessed by qPCR in stage 7 aggregates from control and corrected (*n* = 4–8, black) and patient cells (*n* = 5–7, blue) exposed for 48 hours to vehicle (DMSO), thapsigargin, or tunicamycin. mRNA expression was normalized to the geometric mean of reference genes β-actin and GAPDH. The median is shown by a horizontal line in the box plots; 25th and 75th percentiles are at the bottom and top of the boxes; whiskers represent minimum and maximum values, and data points independent experiments. Comparisons were done by multiple *t* test followed by Bonferroni’s correction for multiple comparisons (**B**), ANOVA followed by Bonferroni’s correction for multiple comparisons (**C** and **D**), and paired-ratio *t* test (**E**). *P < 0.05, **P < 0.01, ***P < 0.001 treatment vs. DMSO; ^#^*P* < 0.05, ^##^*P* < 0.01 vs. control and corrected cells as indicated.

The *YIPF5* patients’ iPSCs differentiated into stage 7 islet cells with somewhat fewer insulin-positive cells and slightly more glucagon-expressing cells compared with healthy control or corrected iPSCs (Figure 6, A and B). The expression of genes during differentiation was comparable between patients’ and control and corrected iPSC lines, with a trend for lower *INS* mRNA expression at stages 6 and 7 (Supplemental Figure 11). The p.(Ile98Ser) mutation did not affect proinsulin and insulin content (Supplemental Figure 12, A and B) nor glucose- and/or forskolin-stimulated insulin secretion (Supplemental Figure 12C).

The viability of *YIPF5* patient β cells was less than that of healthy or corrected β cells (Figure 6C). Similarly, caspase-3/7 activation tended to be higher in patients’ iPSC-β cells compared with corrected β cells (*n* = 2–5; data not shown). Following 48–72 hours of exposure to the ER stressor thapsigargin, tunicamycin, or brefeldin A, *YIPF5*-mutant cells tended to be more prone to undergo apoptosis (Figure 6, C and D). Contrary to the *YIPF5*-KO H1 cells, ER stress signaling was not enhanced by the p.(Ile98Ser) mutation in patient iPSC- or hESC^p.(Ile98Ser)^-β cells under basal condition (Figure 6E). Induction of *CHOP* and *BiP* mRNA expression upon tunicamycin exposure tended to be higher in patients’ β cells compared with healthy control and patient corrected β cells (Figure 6E). In keeping with the results in YIPF5-depleted EndoC-βH1 cells, the proapoptotic BCL-2 family members DP5 and PUMA were induced in ER-stressed *YIPF5*-mutant cells (Figure 6E). Taken together, our results demonstrate that the p.(Ile98Ser) *YIPF5* mutation does not compromise differentiation and function of β cells but affects cell survival by sensitizing them to ER stress–induced apoptosis.

## Discussion

We report 6 patients from 5 families with a congenital syndrome of neonatal/early-onset diabetes, severe microcephaly, and epilepsy caused by biallelic mutations in the *YIPF5* gene. Morphological and functional studies show that *YIPF5* is expressed during human brain development and in adult brain and pancreatic islets and that *YIPF5* deficiency reduces β cell survival by enhancing the ER stress response and sensitizing human β cells to ER stress–induced apoptosis.

The clinical features identified in the 6 patients were very similar, with all of them having severe microcephaly and early-onset epilepsy. All had diabetes, with the age at diagnosis ranging from the neonatal period to early infancy. The disease severity was variable between families, with 5 affected individuals in family II dying in early infancy (the homozygous *YIPF5* mutation could be confirmed only in the proband, as DNA was not available from siblings), while patient I is reported to have normal neuromotor development at the age of 5 years and his epilepsy resolved at the age of 2 years. The missense mutation identified in patient I, p.(Ala181Val), was predicted to be tolerated by 2 of 4 in silico tools used, suggesting the possibility that this variant has a less severe effect on protein function. It is therefore possible that this phenotypic variability is directly linked to the severity of the mutation; however, our cohort of patients with homozygous *YIPF5* mutations is currently too small to allow accurate estimation of any genotype-phenotype relationship.

YIPF5 is a 5-span transmembrane protein (Figure 1B) that cycles between the ER and the Golgi and localizes at ER exit sites, the ER-Golgi intermediate compartment, and part of the *cis*-Golgi (30–33). Previous studies in HeLa cells have suggested that YIPF5 plays a role in cargo exit from the ER (34), resulting in protein overload, thereby triggering the ER stress response. This was consistent with data in yeast reporting delayed ER-to-Golgi transport when a dominant-negative form of the *YIPF5* ortholog *Yip1A* was overexpressed (33). Other studies did not detect delayed anterograde cargo transport when silencing *YIPF5* expression and instead found evidence for a role of YIPF5 in retrograde Golgi-to-ER transport (30, 31). ER whorling and partial Golgi fragmentation have also been observed in in vitro models of YIPF5 depletion, suggesting a role of YIPF5 in ER and Golgi structure maintenance (30, 32–34).

The mutations identified in our patients affect different regions of the YIPF5 protein, 3 of them located in the cytoplasmic domain, which is likely to be important for interactions with other proteins, and the other 2 predicted to affect residues in the transmembrane domains. All these mutations are predicted to be deleterious; however, it is possible that function of the YIPF5 protein is not completely lost. *YIPF5* knockdown in HeLa cells has been previously reported to result in reorganization of the ER into stacked, concentric, whorl-like structures (34). The same group later investigated the effect of a comprehensive range of amino acid substitutions in YIPF5 (35). They reported that even single-residue substitutions of amino acids located in the third and fourth transmembrane domains (which include the p.Ala181 and p.Trp218 residues mutated in families I and IV) severely compromised protein stability. These results thereby support the likely pathogenicity of the p.(Ala181Val) and p.(Trp218Arg) variants we have identified, possibly through impaired protein stability. Dykstra et al. (35) identified 5 YIPF5 residues (3 cytoplasmic and 2 in the transmembrane domain) that, when mutated, resulted in ER whorl formation in HeLa cells. Among the residues investigated were p.Gly97 and p.Ile98, which are mutated in families III and V. Substitutions at these positions were not found to result in whorl formation in the study by Dykstra et al. (35). Consistent with this, we did not observe whorl formation in hESC^p.(Ile98Ser)^-β cells, supporting the previously suggested notion that whorl formation is uncoupled from other YIPF5 functions (35) and our hypothesis that the mutations identified in our patients do not completely abolish YIPF5 function. To try to account for this possibility in our functional experiments, we used a model of complete loss of function (complete KO in hESCs), as well as models of incomplete loss of function [50%–75% knockdown in human EndoC-βH1 cells, iPSCs derived from 2 of the cases with the p.(Ile98Ser) mutation, and hESCs harboring the same homozygous p.(Ile98Ser) mutation].

In all 3 partial loss-of-function models, YIPF5 deficiency resulted in increased expression of ER stress markers upon treatment with the ER stressors thapsigargin, tunicamycin, and brefeldin A, with a tendency to increase apoptosis. In EndoC-βH1 cells, *YIPF5* expression was strongly upregulated by brefeldin A, which blocks ER-to-Golgi transport, suggesting that transcriptional induction of YIPF5 could be part of a compensatory mechanism to overcome the inhibition of trafficking. Further studies will be needed in order to elucidate which pathways regulate YIPF5 expression in response to ER stressors such as brefeldin A and whether Golgi stress response transcription factors such as CREB3 (36) and TFE3 (37) are involved. Taken together, our results support YIPF5’s role in ER-to-Golgi transport and point toward YIPF5 loss sensitizing β cells to ER stress–induced apoptosis. In HeLa cells, YIPF5 has been shown to interact with and promote IRE1 oligomerization and phosphorylation and enhance downstream XBP1 splicing upon tunicamycin exposure or infection with *Brucella abortus* (38). In HeLa and CaSki cells, another human cervical cancer cell line, YIPF5 constitutively activated IRE1 and PERK signaling, with YIPF5-depleted cells showing reduced IRE1 phosphorylation, lower PERK mRNA and protein expression, and less PERK phosphorylation and downstream signaling (39). These contrasting findings suggest that YIPF5 may impact ER stress and the ER stress response in a cell type– and/or context-dependent manner.

As expected, the most striking phenotype was observed when the *YIPF5* gene was completely knocked out in hESCs. These KO β cells showed a strong increase in the proinsulin/insulin ratio, consistent with proinsulin not being transported into the Golgi, and triggered the ER stress response. Furthermore, transmission electron microscopy of YIPF5-KO β cells and α cells showed a pronounced ER distension in the KO β cells resulting from ER stress induced by proinsulin accumulation in the ER. In contrast, the ER in YIPF5-KO α cells was not affected, suggesting that YIPF5 is specifically essential for proinsulin trafficking from the ER in β cells.

The milder phenotype observed in patient-iPSC- and ESC^p.(Ile98Ser)^-derived β cells might indicate that the YIPF5 protein harboring the p.(Ile98Ser) mutation still maintains some residual activity, which is consistent with the 2 patients being diagnosed with diabetes in early infancy (rather than at birth) and having low but measurable C-peptide levels more than 10 years after diabetes diagnosis (Table 1). However, ESC^p.(Ile98Ser)^-derived islet-like aggregates showed a significant decrease in human C-peptide levels associated with increased proinsulin accumulation and signs of ER stress after implantation, demonstrating the evolution of cellular pathology with further maturation of the stem cell–derived β cells. It is possible that the complete absence of YIPF5 protein leads to a more drastic phenotype, as seen in *Yipf5* knockout in mice, which is postnatally lethal (40). In addition, the human *YIPF5* gene appears to be intolerant to loss-of-function variants based on gnomAD gene constraints (pLI = 0.99), further supporting that loss-of-function variants are likely strongly deleterious in humans. More functional experiments and identification of further patients are needed to test these hypotheses.

YIPF5 is widely expressed across tissues (Figure 2A). Our qPCR analysis detected abundant expression in pancreatic tissue, islets, β cells, and brain (Figure 2A). Previous expression studies of swine *YIPF5* showed expression in adipose tissue and spleen, but low expression in intestine, liver, lung, muscle, and kidney; pancreatic tissue was not tested (41). While ubiquitously expressed in human tissues, (partial) *YIPF5* loss of function caused by the mutations results in a β cell– and brain-specific phenotype in the patients, possibly pointing to YIPF5 cargo specificity; it will be of interest to examine this in future studies.

The *YIPF5* expression pattern we observed in human embryonic brain, showing high expression in progenitor and neuronal compartments of the developing cortex in addition to the choroid plexus within the cerebral ventricles, is consistent with *YIPF5* playing a role in neural progenitors and/or neurons during development of the cerebral cortex, which is mostly affected by primary microcephaly. The expression in the choroid plexus is in line with potential control of brain morphogenesis and size, as this structure was recently found to secrete important morphogens and growth factors during embryonic brain development (42, 43). Physiological ER stress controls cortical neurogenesis (44), and sustained ER stress causes microcephaly by perturbing the normal generation and survival of projection neurons during cerebral cortex development (44, 45). As an example, Zika virus infection has been shown to cause microcephaly by inducing ER stress; inhibition of PERK prevents microcephaly in Zika virus–infected mouse embryos (45).

One potential explanation for YIPF5’s essential role in β cell survival is its interaction with components of the COPII vesicle coat protein complex (sec23 and sec24) that mediates COPII-dependent export and the anterograde transport from the ER to the Golgi (33). This pathway plays a vital role in ER homeostasis and β cell survival, with inhibition of Sar1, which initiates COPII assembly, causing alterations in ER morphology and severe ER stress in β cells (46). Consistent with this, ER morphology was severely affected in *YIPF5*-KO hESC-β cells, but we did not observe the whorl-like structures described in *YIPF5*-deficient HeLa cells (34), suggesting that phenotypic specificity may be due to *YIPF5* cell-specific functions. The specific importance of YIPF5 for β cells is further supported by our observation that expression of ER stress markers in islet aggregates derived from *YIPF5*-KO hESCs was limited to the β cells, while α cells had normal glucagon expression and no ER stress. These data strongly suggest that while β cells are highly dependent on YIPF5 function, α cells appear to be able to survive without YIPF5.

While disruption of ER-to-Golgi trafficking is known to result in at least 10 different neurological disorders (47), its involvement in the etiology of diabetes has been less clear. Recently a truncating mutation in the *TANGO1* (*MIA3*) gene, encoding a protein involved in the export of bulky cargos from the ER to the Golgi, has been reported in one consanguineous family with a complex syndrome of dentinogenesis imperfecta, short stature, skeletal abnormalities, sensorineural hearing loss, and mild intellectual disability. All 4 affected individuals also had insulin-dependent diabetes, highlighting the importance of the mechanisms regulating cargo exit from the ER for β cell function (48). A β cell–specific knockout of cTAGE5, a TANGO1-interacting protein, has been previously shown to impair proinsulin trafficking, induce ER stress, and cause impaired glucose tolerance in mice (49). Furthermore, impaired ER-to-Golgi trafficking may play a role in β cell dysfunction and death caused by environmental insults in type 2 diabetes, as exposure of β cells to the saturated free fatty acid palmitate reduces protein trafficking, thereby contributing to ER stress (19). The molecular mechanisms by which palmitate exerts these effects remain to be fully elucidated.

In conclusion, we report homozygous mutations in *YIPF5* as the genetic cause of an autosomal recessive syndrome characterized by microcephaly, epilepsy, and neonatal/early-onset diabetes. Functional studies show that *YIPF5* deficiency affects β cell function by enhancing ER stress and sensitizing human β cells to ER stress–induced apoptosis. To the best of our knowledge, this is the first report of mutations in a gene affecting ER-to-Golgi trafficking resulting in diabetes by increasing β cell ER stress, uncovering a critical role of YIPF5 in the human β cell. Our findings highlight a biological pathway essential for β cells.

## Methods

Further information can be found in Supplemental Methods.

### Patient cohort.

Patients with neonatal diabetes were recruited by their clinicians for molecular genetic analysis in the Exeter Molecular Genetics Laboratory.

### Genetic analysis.

Genome sequencing was performed on DNA extracted from peripheral blood leukocytes of 2 probands diagnosed with neonatal diabetes, microcephaly, and epilepsy. Samples were sequenced on an Illumina HiSeq 2500 with a mean read depth of 38.3 for patient I and 33.6 for patient II. The sequencing data were analyzed using an approach based on the GATK best practice guidelines. GATK HaplotypeCaller (https://gatk.broadinstitute.org/hc/en-us/articles/360037225632-HaplotypeCaller) was used to identify variants that were annotated using Alamut batch version 1.8 (Interactive Biosoftware), and variants that failed the QD2 VCF filter or had less than 5 reads supporting the variant allele were excluded. Copy number variants were called by SavvyCNV, which uses read depth to judge copy number states. SavvyVcfHomozygosity was used to identify large (>3 Mb) homozygous regions in the genome sequencing data (https://github.com/rdemolgen/SavvySuite).

Replication studies were performed in a cohort of 187 patients diagnosed with diabetes before age 12 months in whom the known genetic causes of neonatal diabetes had been excluded. Patients were analyzed using a targeted next-generation sequencing assay (50), which includes baits for known neonatal diabetes genes and additional candidate genes followed up from gene discovery, such as *YIPF5*, or by independent exome sequencing analysis. Variant confirmation and cosegregation in family members were performed by Sanger sequencing (see Supplemental Table 4 for primer sequence).

The bioinformatics tools SIFT, PolyPhen-2, MutationTaster, and Align GVGD were accessed through the Alamut software (Interactive Biosoftware) to predict the effect of variants on the YIPF5 protein.

### Human β cell and islet culture and treatment.

Human insulin-producing EndoC-βH1 cells, provided by R. Scharfmann (Institut Cochin, Université Paris Descartes, Paris, France), were cultured as previously described (51, 52). EndoC-βH1 cells were exposed to 1 μM thapsigargin or 0.05 μg/mL brefeldin A in medium containing 2% FBS. All compounds were from Sigma-Aldrich. The vehicle DMSO was added to the control condition in all experiments.

Human islets from nondiabetic organ donors (*n* = 4, 2 female and 2 male donors; age 62 ± 9 years; BMI 27 ± 3 kg/m^2^; cause of death: 3 cerebral hemorrhage, 1 cardiovascular disease) were isolated by collagenase digestion and density gradient purification, and cultured as previously described (53). The percentage of β cells of the human islet preparations was 59% ± 5%, as determined by insulin immunofluorescence.

### RNA interference.

YIPF5 was silenced using 2 siRNAs targeting different sequences of YIPF5 (si1 SI04182745 and si2 SI04344984, Qiagen). CHOP was silenced using SI3041633 (Qiagen) and DP5 using s194952 (Ambion). Allstar Negative Control siRNA (siCT, Qiagen) was used as negative control. This siRNA does not affect β cell gene expression, function, or viability (54). Transient transfection was performed using 30 nM siRNA and Lipofectamine RNAiMAX lipid reagent (Invitrogen/Life Technologies) as previously described (54). After overnight transfection, cells were cultured at least 8 hours before treatment.

### RNA in situ hybridization.

In situ hybridization on human fetal brain tissue was performed using digoxigenin-labeled RNA probes (DIG RNA labeling kit, Roche) as previously described (55). The riboprobe template was designed to target the first 764 nucleotides of the 6 different splice variants of human YIPF5. The template was amplified by PCR using human YIPF5-specific primers: forward 5′-ggtggt**gagctc**ATGCTGGCTATGACTAATC-3′, reverse 5′-ggtggt**ggtacc**AAATCTGCATGAGAG-3′, in which SacI and KpnI restriction sites (highlighted in bold) were added to allow the directional cloning of the probe into pBluescript II SK(+) plasmid. Sense and antisense probes were generated by transcription of the KpnI or SacI linearized plasmid with T3 or T7 RNA polymerases, respectively.

### Genome editing of hESCs and iPSCs.

For knocking out the *YIPF5* gene in H1 hESCs, the third exon was deleted using 2 CRISPR/Cas9 guides that were designed with Benchling (Biology Software, 2019) (G1.1 GGCTATGACTATTCGCAGCA and G1.2 GATGAGCCACCTTTATTAGA). The ribonucleoprotein (RNP) components (HiFi Cas9 protein, crRNA and tracrRNA) were purchased from Integrated DNA Technologies (IDT) and prepared based on the manufacturer’s protocol. Two million cells were electroporated with the RNP complex using Neon Transfection System (1100 V, 20 milliseconds, 2 pulses, Thermo Fisher Scientific) and single-cell-cloned using limiting dilution. The sequence of the primers used to screen the formed colonies is provided in Supplemental Table 5. The KO cell line was characterized for pluripotency using immunocytochemistry for OCT4, TRA1-60, and SSEA4, and for gene expression levels of *OCT4*, *SOX2*, and *NANOG* by qPCR.

CRISPR/Cpf1 was used to correct the mutation p.(Ile98Ser) in the patient iPSC line ULBi006.SA7 and to introduce the mutation p.(Ile98Ser) in WT H1 hESCs using homology-directed repair (HDR). An isogenic control for the patient iPSCs was generated using a 21-base guide, CCAGATGTGGTCAAAATTGCT, while the guide for introducing the mutation in H1 was CCAGATGTGGTCAAAATTGAT. The correction and mutation templates were generated as a 200-bp PCR amplicon using 110-base forward and reverse primers (see Supplemental Table 5 for sequence).

The templates were designed to introduce silent mutations to generate a restriction site to facilitate the screening of the clones using restriction enzymes. The restriction site for the correction template was PfeI, while BamHI was created for the mutation template. The RNP components (Alt-R A.s. Cpf1 Ultra and crRNA) were purchased from IDT and prepared based on the manufacturer’s protocol. The cells were electroplated as mentioned before with 2 μg HDR template, then single-cell-sorted and screened using PCR (see Supplemental Table 5 for primer sequence), followed by enzyme restriction. Positive clones were confirmed using Sanger sequencing at Eurofins Genomics, and the sequences were aligned using Geneious Prime 2020.1.1. The top 3 off-target hits predicted by the online tool CRISPOR (56) were checked, and no off-target indels were found (see Supplemental Table 5 for primer sequence).

### hESC culturing, differentiation into β cells, and chemical ER-stress induction.

The H1 hESCs were obtained from WiCell, Wisconsin Materials [provider scientist, Maike Sander, University of California; stock WA01 (H1)]. Differentiation of hESCs to pancreatic endocrine cells was performed using published protocols (57–59) with further modifications. Cells were seeded at a density of 1.8 million cells per 3.5 cm well on Matrigel-coated plates with E8 medium containing 10 μM ROCK inhibitor. Differentiation was started 24 hours later and proceeded through a 7-stage differentiation protocol (stages 1–4 in adherent culture, stage 5 in AggreWell [34421, Stemcell Technologies], and stages 6 and 7 in suspension culture). After 1 week of stage 7, 50 hESC-derived islet-like aggregates were transferred into a 12-well plate in 1 mL of stage 7 medium with brefeldin A (B5936, Sigma-Aldrich) at 1 μg/mL for 24 hours, thapsigargin (T9033, Sigma-Aldrich) and tunicamycin (T7765, Sigma-Aldrich) at 1 μM and 5 μg/mL, respectively, for 48 hours. DMSO was used as a vehicle control at 5 μL/mL. Aggregates were PFA-fixed for immunohistochemistry.

### Transplantation of differentiated cells.

NOD/SCID-γ mice (005557, The Jackson Laboratory) were obtained from SCANBUR and housed at Biomedicum Helsinki animal facility, on a 12-hour light/12-hour dark cycle and food ad libitum. Transplantations were performed on 3- to 9-month-old mice as described previously (60). Briefly, aggregates equivalent to approximately 3 million cells were loaded on PE-50 tubing and transplanted under the kidney capsule. Mice were anesthetized with isoflurane. Carprofen (5 mg/kg, subcutaneously; Rimadyl, Pfizer, Helsinki, Finland) and buprenorphine (0.05–0.1 mg/kg, subcutaneously; Temgesic, RB Pharmaceuticals Ltd.) were used as analgesics during the operation and the following day. Mouse blood samples were collected monthly from the saphenous vein using heparinized capillary tubes. Plasma was separated by centrifugation at 5000 RCF for 5 minutes at room temperature.

### PBMC reprogramming into iPSCs and iPSC quality control.

PBMCs from patients IIIa and IIIb were reprogrammed into iPSCs using Sendai virus. Cells were plated in RPMI with 10% FBS (Gibco) at 10^6^ cells per well of a 6-well low-attachment plate (3471, Corning) and infected the next day with SeVdp (KOSM302L) vector (22MAT1411, National Institute of Advanced Industrial Science and Technology, Tokyo, Japan) at MOI 2 for 2 hours. Medium was refreshed with E6 medium (Gibco), and cells were transferred to Matrigel-coated plates (Corning BV, Life Sciences). From day 8, cells were cultured in E8 medium (Life Technologies) and medium was changed every second day. Emerging iPSC colonies were manually picked up. Vector removal was confirmed by reverse-transcriptase PCR with the following primers: 5′-AGACCCTAAGAGGACGAAGACAGA-3′ and 5′-ACTCCCATGGCGTAACTCCATAG-3′. For the embryoid body assay, iPSCs reaching 60%–70% confluence were detached with 0.5 mM EDTA (Life Technologies), resuspended in E8 medium containing 10 μM ROCK inhibitor (Stemcell Technologies), and transferred to super-low-attachment plate (Corning) on a rotating platform to form aggregates. The next day, embryoid bodies were resuspended in DMEM/F-12 medium (Gibco) containing Glutamax (Gibco), 10% KSR (Life Technologies), 1% NEAA (Thermo Fisher Scientific), 0.1 mM β-mercaptoethanol (Gibco), and 1% penicillin/streptomycin. Medium was changed every second day for 1 week. Embryoid bodies were plated on Matrigel-coated ICC chambers (15–20 per well) for 2 weeks, with medium refreshed every second day, and fixed in PFA 4% for immunocytochemistry. For karyotyping, KaryoMax Colcemid solution (Gibco 15210) was added to 80% confluent iPSCs at a final concentration of 500 ng/mL for 3–4 hours at 37°C. Cells were washed with PBS, incubated in Trypsin/EDTA solution (R001100, Thermo Fisher Scientific) at room temperature for 2 minutes, detached, collected in DMEM/F-12 with 10% FBS (Gibco), centrifuged for 10 minutes at 300 *g*, and resuspended in 0.0075 M KCl. After 10 minutes of incubation, cells were centrifuged at 600 *g* for 10 minutes and resuspended in the fixative methanol/acetic acid 3:1 (Merck). After 20 minutes, cells were washed twice with the fixative and stored at 4°C. Karyotyping was done at the Institute of Pathology and Genetics, Gosselies, Belgium.

### iPSC culturing and differentiation into β cells.

iPSCs were cultured in Matrigel-coated plates (Corning) in E8 medium as previously described (61, 62). Four *YIPF5* mutant cell lines (2 clones from each patient, namely, ULBi006.SA2 and ULBi006.SA7, ULBi007.BA2 and ULBi007.BA11) were used as well as a previously characterized control cell line (HEL115.6) (63). The differentiation into β cells was done as previously described (62, 63). Cells were seeded at 2.5 × 10^6^ cells per 3.5 cm well in E8 medium containing 5 μM ROCK inhibitor (Stemcell Technologies). Differentiation was started 24 hours later. Until the pancreatic progenitor stage (stage 4), cells were differentiated in Matrigel-coated wells, after which they were plated in microwell plates at 900 cells per microwell (AggreWell) to form islet-like aggregates. The differentiation was continued in microwells. After 1 week of stage 7, aggregates were exposed to 1 μM thapsigargin, 5 μg/mL tunicamycin, or 0.01 μg/mL brefeldin A in stage 7 medium. DMSO was used as vehicle control.

### Insulin content and secretion.

EndoC-βH1 cells were preincubated in DMEM containing 2.8 mM glucose for 24 hours, followed by incubation in glucose-free Krebs solution for 1 hour. Cells were then exposed to Krebs containing 0 mM or 20 mM glucose or 20 mM glucose plus 10 μM forskolin for 40 minutes. Insulin was measured in cell-free supernatants and acid-ethanol–extracted cell lysates, the latter normalized for total protein content measured by Bradford dye method. Fifty hESC-derived stage 7 aggregates were washed twice with glucose-free Krebs buffer and preincubated on a rotating platform with 1 mL 3.3 mM glucose-containing Krebs buffer for 1 hour. Aggregates were incubated sequentially in 3.3 mM glucose, 20 mM glucose, and 3.3 mM plus 30 mM KCl for periods of 30 minutes. Supernatants were stored at −80°C for ELISA. Aggregates were lysed by sonication and acid-ethanol for determination of insulin and DNA contents. Insulin secretion results are presented as insulin released after cell mass normalization using DNA content of the β cell percentage assessed by FACS. Forty iPSC-derived stage 7 aggregates were washed with glucose-free Krebs buffer (Univercell Biosolutions) in low-adhesion plates (83.1836, Starstedt) and preincubated in 1.6 mM glucose Krebs for 30 minutes. Aggregates were exposed to 1.6 mM glucose, 16.7 mM glucose, or 16.7 mM glucose plus 10 μM forskolin for 1 hour. Hormone content was normalized for total protein content.

### ELISA.

Human C-peptide, proinsulin, and insulin levels were measured from plasma samples and cell supernatants and lysates with Ultrasensitive C-peptide, proinsulin, and insulin ELISAs (all from Mercodia, Sweden) according to the manufacturer’s instructions.

### Statistics.

Statistical analyses were performed with GraphPad Prism (version 7.0c, GraphPad Software). Data points represent independent experiments. In secretion experiments, data points represent the average of biological duplicates. In the box plots, the median is shown by a horizontal line; 25th and 75th percentiles are at the bottom and top of the boxes; whiskers represent minimum and maximum values. In time course experiments, data are shown as mean ± SEM. For the EndoC-β1 and iPSC experiments, comparisons between groups were performed by paired 2-way ANOVA or mixed-model analysis (in case of a missing value), followed by 2-tailed *t* tests with the Bonferroni correction for multiple comparisons. For hESC experiments, the parametric unpaired 2-tailed *t* test and 1-way and 2-way ANOVA tests with the Bonferroni multiple-comparisons test were used to compare the sum of ranks. *P* values less than 0.05 were considered statistically significant.

### Study approval.

The genetic study in the Exeter Molecular Genetics Laboratory was conducted in accordance with the Declaration of Helsinki, and all patients or their parents gave informed consent for genetic testing.

Human pancreata not suitable for clinical purposes were collected from nondiabetic, brain-dead organ donors after written informed consent from next of kin, and handled as described (64) with the approval of the Ethical Committee, University of Pisa.

The human fetal brain study was approved by the Ethics Committee of Erasmus Hospital, Université Libre de Bruxelles. Written informed consent was given by the parents.

Mouse experiments were approved by the National Animal Experiment Board in Finland (ESAVI/14852/2018).

Patients’ PBMCs were obtained after written informed consent was given by patients or parents with approval by the Erasmus Hospital Ethics Committee (ref. P2008/313). Written informed consent for inclusion of patient photographs in scientific publications was collected by the referring clinicians from one parent of each patient.

## Author contributions

EDF, ATH, SEF, SE, TO, and MC conceived the project. EDF, ML, HI, HM, FF, JSV, ATH, MC, TO, PM, and DLE planned the experiments. EDF, MBJ, SEF, CJ, VS, and KP analyzed the genetic data. MNW and TWL performed bioinformatics analysis for genome sequencing and targeted next-generation sequencing data. VL planned and conducted morphometric analyses. HV and EJ performed the electron microscopic analyses. ATH, BH, MNO, EU, RY, TG, MY, KP, and BA analyzed the clinical data. CD, YC, CC, TS, HS, and NP performed experiments in EndoC-βH1 cells and iPSCs-derived β cells. YC reprogrammed patient PBMCs into iPSCs. MIE, AB, IS, and PV performed in situ hybridization. EDF, ML, and HI wrote the first draft of the manuscript. All authors reviewed and improved the manuscript. The assignment of the authorship order for the first 6 authors is mainly chronological and reflects the equal contributions from the 3 research groups: EDF identified the causative gene in patients with neonatal diabetes and microcephaly, ML performed the first functional studies suggesting that ER stress may be the causative mechanism, HI and HM generated the genetically modified models and performed their functional studies, HI also generated the mutation correction in patient-derived iPSCs, MNW developed the genome sequencing and homozygosity mapping pipelines that allowed identification of the causative gene, and FF performed studies in patient-derived iPSCs.

## Supplementary Material

Supplemental data

## Figures and Tables

**Table 1:**
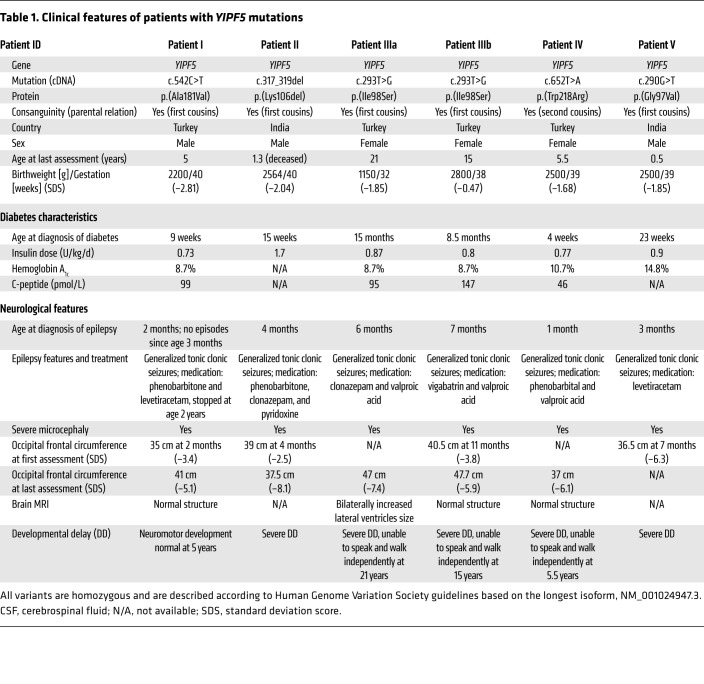
Clinical features of patients with *YIPF5* mutations

## Version 1 11/09/2020

Electronic publication

## Version 2 12/01/2020

Print issue publication
