# Supplementary material for: YIPF5 mutations cause neonatal diabetes and microcephaly through endoplasmic reticulum stress
Source: J Clin Invest. 2020 Nov 9;130(12):6338–53. doi: 10.1172/JCI141455 (PMC7685733; doi:10.1172/JCI141455)
Supplement: Supplemental data [file jci-130-141455-s463.pdf]

Supplementary appendix

YIPF5 mutations cause neonatal diabetes and microcephaly through endoplasmic reticulum stress

Elisa De Franco*, Maria Lytrivi*, Hazem Ibrahim*, Hossam Montaser*, Matthew Wakeling*, Federica Fantuzzi*, Kashyap Patel, Céline Demarez, Ying Cai, Mariana Igoillo-Esteve, Cristina Cosentino, Väinö Lithovius, Helena Vihinen, Eija Jokitalo, Thomas W Laver, Matthew B Johnson, Toshiaki Sawatani, Hadis Shakeri, Nathalie Pachera, Belma Haliloglu, Mehmet Nuri Ozbek, Edip Unal, Ruken Yıldırım, Tushar Godbole, Melek Yildiz, Banu Aydin, Angeline Bilheu, Ikuo Suzuki, Sarah E Flanagan, Pierre Vanderhaeghen, Valérie Senée, Cécile Julier, Piero Marchetti, Decio L Eizirik, Sian Ellard, Jonna Saarimäki-Vire, Timo Otonkoski*, Miriam Cnop*, Andrew T Hattersley*

***Contributed equally**

Supplemental Materials and Methods

Western blot

Cells were lysed in Laemmli buffer, extracts resolved on 10–14% SDS-PAGE and transferred to nitrocellulose membrane (Bio-Rad). Immunoblotting was performed by overnight incubation with antibodies against YIPF5 and GAPDH, the latter used as control for protein loading. After incubation with secondary horseradish peroxidase-conjugated antibody, proteins were detected using SuperSignal West Femto chemiluminescence revealing reagent (ThermoFisher) in a ChemiDoc XRS+ system and quantified by Image Lab software (Bio-Rad). The antibodies and dilutions used are provided in Supplementary Table 9.

Human fetal tissue collection and preparation

Human fetuses were obtained following medical pregnancy termination. Fetuses aged 12-18-21 gestational weeks were used for in situ hybridization of brain tissue and processed as previously described (1). All cases were examined with standard feto-pathological procedures and none displayed clinical or neuropathological evidence of brain malformation. As soon as possible after expulsion (less than 6 hours), the brain was removed using the standard fetal autopsy procedure, frozen in liquid nitrogen for RNA extraction and embedded as a whole in OCT compound (Tissue-Tek Sakura, VWR Cat# 4583), then snap-frozen in a 2-methylbutane on dry ice bath for histological studies.

Flow cytometry analysis

For quantifying the definitive endoderm positive cells in stage 1, cytometry for CXCR4 was performed as previously described (2). For intracellular antigen cytometry of stage 7, cells were dissociated with TrypLE for 6 min at 37°C and resuspended in cold 5% FBS-containing PBS. Cells were fixed and permeabilized using Cytofix/Cytoperm (554714, BD Biosciences) as recommended by manufacturer. Primary or conjugated antibodies were incubated with the cells overnight at 4°C in Perm/Wash buffer (554714, BD Biosciences) containing 4% FBS. Cells were washed twice with Perm/Wash buffer and analysed using FACSCalibur cytometer (BD Biosciences) and FlowJo software (Tree Star Inc.). Details of the antibodies used for FACS analysis are given in Supplementary Table 6.

Transmission electron microscopy

Samples were fixed using 2.5% glutaraldehyde (EM-grade, Sigma-Aldrich) in 0.1 M sodium cacodylate buffer, pH 7.4, supplemented with 2 mM calcium chloride, at RT for 2h. After washing, the samples were post-fixed with (non-reduced) 1% osmium tetroxide (Electron Microscopy Sciences) in the same buffer supplemented with 2 mM calcium chloride, at RT for 1h. Samples were then dehydrated in ethanol series and acetone prior to gradual infiltration into Epon (TAAB 812) over 28h and polymerization at 60°C o/n. Ultrathin 60-nm-thick sections were cut, picked on Pioloform coated single slot grids and post-stained with uranyl acetate and lead citrate. Sections were examined with a Hitachi HT7800 microscope (Hitachi High-Technologies) operated at 100 kV and images were acquired using a Rio9 CMOS-camera (Gatan Inc., AMETEK, Pleasanton, CA). Cells for analysis from two differentiation experiments were selected based on the characteristics features of mature granules and images for α-cells and β-cells were collected with random systematic sampling: 3-5 images per cell, 4 cells per aggregate and 5 aggregates per sample. ER morphology was evaluated from the micrographs acquired at nominal magnification of 5,000X.

mRNA extraction and qPCR

Poly(A)+-RNA was isolated from EndoC-βH1 cells, human islets and iPSC-derived cells using Dynabeads mRNA DIRECT kit (Invitrogen), and reverse transcribed. The real-time qPCR amplification was performed on CFX Connect (Bio-Rad) or Rotor-Gene Q (Qiagen) instruments and compared to a standard curve. Expression values were normalized to the reference genes β-actin (ACTB), glyceraldehyde 3-phosphate dehydrogenase (GAPDH) and/or ornithine decarboxylase antizyme 1 (OAZ1).

Total RNA from ESC-derived cells was isolated using NucleoSpin Plus RNA kit (Macherey-Nagel). 1.5 µg RNA was reverse transcribed using Moloney murine leukemia virus reverse transcriptase (M1701, Promega) for 90 min at 37°C. 50 ng cDNA was amplified using 5x HOT FIREPol EvaGreen qPCR Mix Plus (no ROX) in a 20 µL reaction. The reactions were pipetted using QIAgility (Qiagen) robot into 100 well disc run in Rotor-Gene Q. Relative quantification of gene expression was analyzed using ΔΔCt method, with cyclophilin G (PPIG) as reference gene.

Reverse transcription without template was used as negative control and exogenous positive control was used as a calibrator. qPCR primer sequences are provided in Supplementary tables 7 and 8.

Assessment of apoptosis

The percentage of viable, apoptotic and necrotic cells was determined by fluorescence microscopy after 15 min incubation with the DNA-binding dyes propidium iodide (5 µg/mL) and Hoechst 33342 (10 µg/mL) (Sigma-Aldrich). A minimum of 400 EndoC-βH1 cells was counted for each experimental condition by two observers, one of them unaware of sample identity. The agreement between researchers was >90%. This fluorescence assay for single cells is quantitative and has been validated by comparison against electron microscopy, DNA ladder formation and caspase 3/9 activation (3). The RealTime-Glo Annexin V Apoptosis and Necrosis Assay (Promega) was used to detect phosphatidylserine on the outer cell membrane using two annexin V fusion proteins coupled to complementary luciferase subunits. Luminescence was measured with a Victor 3 plate reader at baseline (30 min-1h after addition of reagents) and at subsequent time points that were normalized to baseline. Background luminescence of no-cell wells was subtracted from each measurement. For TUNEL assay, paraffin sections were processed with In Situ Cell death Detection Fluorescein kit (Roche, #11684795910) according to manufacturer's instructions.

Immunocytochemistry and immunohistochemistry

For paraffin embedding, stage 7 aggregates were fixed with 4% PFA at room temperature for 3h, and then stained with eosin. Afterwards, aggregates were embedded in 2% low-melting agarose (Fisher Bioreagents) and transferred to paraffin blocks. ESC grafts were retrieved at 1 or 3 months, dissected and fixed with 4% PFA at room temperature for 48h, paraffin embedded, and blocks cut into 5 µm sections using Leica microtome. For immunohistochemistry, slides were deparaffinized and antigens retrieved by boiling slides in 0.1 M citrate buffer (pH 6) using Decloaking chamber (Biocare Medical) at 95°C for 12 min. Immunofluorescence staining was imaged with Zeiss Axio Observer Z1 with Apotome two and processed with ZEN2 software blue edition. Paraffin sections were stained simultaneously and imaged on the same session with the same microscope parameters. For single

cell staining, stage 7 aggregates were dissociated after 2 washes with 0.5 mM EDTA (Life Technologies). Accumax (Sigma Aldrich #A7089) was added and cells were incubated for 4 min at 37°C, gently shaken, and incubated for 4 additional min at 37°C. Cells were resuspended in KSR (Life Technologies #10828010) containing 10 µM ROCK inhibitor (StemCell technologies) and gently pipetted. Dissociated cells were resuspended in stage 7 medium containing 10 µM ROCK inhibitor and plated on Matrigel-coated ICC chambers. After 12h, cells were washed once with PBS, fixed in 4% PFA for 20 min, permeabilized with 0.5% triton-X100 in PBS for 10 min, washed with PBS, blocked with UltraV block (Thermofisher) for 10 min, all at room temperature, and incubated overnight at 4°C with primary antibodies diluted in 0.1% Tween in PBS. Following incubation with secondary antibodies for 45 min at room temperature samples were mounted with Vectashield with DAPI (Vector Laboratories) and covered with glass coverslips. Antibodies are listed in Supplementary Table 9.

Morphometric analysis

Image quantifications were performed using custom CellProfiler 3.0 pipelines (4). For the analysis of insulin or proinsulin staining intensity, the nuclei were identified first, and cytoplasmic objects were created by adjusting the intensity gradient of the background signal. A higher threshold was then applied to localize the individual hotspots of insulin and proinsulin immunoreactivity. The hotspots were summed together in each cytoplasm and compared to the total area of that cytoplasm at a single cell basis. Images of insulin/BiP and insulin/glucagon co-immunostaining were analysed with a higher threshold to only detect the specific immunoreactive signals which were then compared to the adjacent nuclei. If 15% of the perimeter of a nucleus was surrounded by cytoplasm positive for insulin, glucagon or BiP, the cell was considered to be positive for that cytoplasmatic marker.

Supplementary table 1 – Homozygous rare (MAF<0.001) variants with at least 5 supporting reads identified in Case I and percentage of homozygosity as calculated from genome sequencing data.

Gene	Coding Effect	Allele depth	gNomenclature	cNomenclature	pNomenclature
TIE1	missense	0,31	Chr1(GRCh37):g.43770797C>T	NM_001253357.1:c.199C>T<br>NM_005424.4:c.334C>T	NM_001253357.1:p.Arg67Trp<br>NM_005424.4:p.Arg112Trp
CFH	missense	0,43	Chr1(GRCh37):g.196697521T>G	NM_000186.3:c.2282T>G	NM_000186.3:p.Leu761Arg
TRAK1	in-frame	0,24	Chr3(GRCh37):g.42251607_42251609del	NM_001265608.1:c.2093_2095del<br>NM_001265609.1:c.1871_1873del<br>NM_014965.4:c.1919_1921del	NM_001265608.1:p.Glu698del<br>NM_001265609.1:p.Glu624del<br>NM_014965.4:p.Glu640del
C1QTNF7	missense	0,33	Chr4(GRCh37):g.15444178G>A	NM_001135171.1:c.625G>A<br>NM_031911.4:c.625G>A<br>NM_001135170.1:c.646G>A	NM_001135171.1:p.Gly209Arg<br>NM_031911.4:p.Gly209Arg<br>NM_001135170.1:p.Gly216Arg
PCDHA7	missense	0,45	Chr5(GRCh37):g.140215746A>G	NM_018910.2:c.1778A>G	NM_018910.2:p.Lys593Arg
YIPF5	**missense**	**0,41**	**Chr5(GRCh37):g.143541851G>A**	**NM_001024947.3:c.542C>T**<br>**NM_001271732.1:c.380C>T**<br>**NM_030799.8:c.542C>T**	**NM_001024947.3:p.Ala181Val**<br>**NM_001271732.1:p.Ala127Val**<br>**NM_030799.8:p.Ala181Val**
FDFT1	in-frame	0,37	Chr8(GRCh37):g.11666252_11666257del	NM_001287750.1:c.226_231del	NM_001287750.1:p.His76_Ser77del
VPS37C	missense	0,36	Chr11(GRCh37):g.60901670G>C	NM_017966.4:c.103C>G	NM_017966.4:p.Leu35Val
ATG2A	missense	0,35	Chr11(GRCh37):g.64676836A>G	NM_015104.2:c.2111T>C	NM_015104.2:p.Ile704Thr
CABP2	stop gain	0,37	Chr11(GRCh37):g.67290170G>T	NM_001318496.1:c.74C>A	NM_001318496.1:p.Ser25*
KRTAP5-7	in-frame	0,13	Chr11(GRCh37):g.71238682_71238741dup	NM_001012503.1:c.336_395dup	NM_001012503.1:p.Gln113_Cys132dup
BPTF	missense	0,5	Chr17(GRCh37):g.65822127G>C	NM_004459.6:c.287G>C<br>NM_182641.3:c.287G>C	NM_004459.6:p.Gly96Ala<br>NM_182641.3:p.Gly96Ala
ZNF431	missense	0,44	Chr19(GRCh37):g.21366801A>C	NM_001319124.1:c.1698A>C<br>NM_001319126.1:c.1422A>C<br>NM_001319127.1:c.1374A>C<br>NM_133473.3:c.1695A>C	NM_001319124.1:p.Glu566Asp<br>NM_001319126.1:p.Glu474Asp<br>NM_001319127.1:p.Glu458Asp<br>NM_133473.3:p.Glu565Asp
FUZ	missense	0,47	Chr19(GRCh37):g.50312016C>G	NM_001171937.1:c.743G>C<br>NM_025129.4:c.851G>C	NM_001171937.1:p.Arg248Pro<br>NM_025129.4:p.Arg284Pro
ECH1	missense	0,29	Chr19(GRCh37):g.39306949C>G	NM_001398.2:c.706G>C	NM_001398.2:p.Glu236Gln
FCGBP	missense	0,35	Chr19(GRCh37):g.40411823C>T	NM_003890.2:c.3805G>A	NM_003890.2:p.Asp1269Asn
SLC22A8	missense	0,37	Chr11(GRCh37):g.62763203C>T	NM_004254.3:c.974G>A<br>NM_001184733.1:c.701G>A<br>NM_001184736.1:c.605G>A<br>NM_001184732.1:c.974G>A	NM_004254.3:p.Arg325His<br>NM_001184733.1:p.Arg234His<br>NM_001184736.1:p.Arg202His<br>NM_001184732.1:p.Arg325His
RAD54L	missense	0,36	Chr1(GRCh37):g.46743781C>T	NM_001142548.1:c.2071C>T<br>NM_003579.3:c.2071C>T	NM_001142548.1:p.Arg691Trp<br>NM_003579.3:p.Arg691Trp
IL17REL	missense	0,48	Chr22(GRCh37):g.50439556C>T	NM_001001694.2:c.64G>A	NM_001001694.2:p.Gly22Ser
ADGRA3	missense	0,30	Chr4(GRCh37):g.22389464T>C	NM_145290.3:c.3830A>G	NM_145290.3:p.Gln1277Arg
CMYA5	missense	0,24	Chr5(GRCh37):g.79029836G>A	NM_153610.4:c.5248G>A	NM_153610.4:p.Gly1750Arg
ANKRD34B	missense	0,34	Chr5(GRCh37):g.79854793G>A	NM_001004441.2:c.1046C>T	NM_001004441.2:p.Thr349Ile
CCDC124	missense	0,33	Chr19(GRCh37):g.18054511A>G	NM_138442.3:c.659A>G<br>NM_001136203.1:c.659A>G	NM_138442.3:p.Asn220Ser<br>NM_001136203.1:p.Asn220Ser
CFAP57	missense	0,31	Chr1(GRCh37):g.43688634G>A	NM_001195831.2:c.2672G>A	NM_001195831.2:p.Arg891Gln
ZNHIT2	missense	0,39	Chr11(GRCh37):g.64884131T>C	NM_014205.3:c.995A>G	NM_014205.3:p.His332Arg
OR2T11	missense	0,51	Chr1(GRCh37):g.248789528A>G	NM_001001964.1:c.902T>C	NM_001001964.1:p.Val301Ala
FBXO38	missense	0,55	Chr5(GRCh37):g.147807003A>G	NM_030793.4:c.2146A>G	NM_030793.4:p.Ser716Gly
DLL3	missense	0,41	Chr19(GRCh37):g.39994735C>G	NM_016941.3:c.677C>G<br>NM_203486.2:c.677C>G	NM_016941.3:p.Pro226Arg<br>NM_203486.2:p.Pro226Arg

Supplementary table 2 – Homozygous rare (MAF<0.001) variants with at least 5 supporting reads identified in Patient II and percentage of homozygosity as calculated from genome sequencing data.

Gene	Coding Effect	Allele depth	gNomenclature	cNomenclature	pNomenclature
ENG	missense	0,29	Chr9(GRCh37):g.130588920G>A	NM_000118.2:c.392C>T<br>NM_000118.3:c.392C>T<br>NM_001114753.1:c.392C>T<br>NM_001114753.2:c.392C>T	NM_000118.2:p.Pro131Leu<br>NM_000118.3:p.Pro131Leu<br>NM_001114753.1:p.Pro131Leu<br>NM_001114753.2:p.Pro131Leu
FBN2	missense	0,35	Chr5(GRCh37):g.127597441G>A	NM_001999.3:c.8351C>T	NM_001999.3:p.Pro2784Leu
KCNN3	in-frame	0,7	Chr1(GRCh37):g.154842200_154842211dup	NM_002249.5:c.230_241dup<br>NM_001204087.1:c.230_241dup	NM_002249.5:p.Gln77_Gln80dup<br>NM_001204087.1:p.Gln77_Gln80dup
PPAT	missense	0,41	Chr4(GRCh37):g.57268336A>T	NM_002703.4:c.673T>A	NM_002703.4:p.Ser225Thr
PCDHA6	missense	0,35	Chr5(GRCh37):g.140207942G>A	NM_031848.2:c.266G>A	NM_031848.2:p.Arg89Gln
YIPF5	**in-frame**	**0,34**	**Chr5(GRCh37):g.143543785_143543787del**	**NM_001024947.3:c.317_319del**<br>**NM_001271732.1:c.155_157del**<br>**NM_030799.8:c.317_319del**	**NM_001024947.3:p.Lys106del**<br>**NM_001271732.1:p.Lys52del**<br>**NM_030799.8:p.Lys106del**
SPINK5	missense	0,36	Chr5(GRCh37):g.147468154A>G	NM_001127698.1:c.460A>G<br>NM_001127699.1:c.460A>G<br>NM_006846.3:c.460A>G	NM_001127698.1:p.Ser154Gly<br>NM_001127699.1:p.Ser154Gly<br>NM_006846.3:p.Ser154Gly
CDCA7L	missense	0,44	Chr7(GRCh37):g.21942673G>C	NM_001127370.2:c.1155C>G<br>NM_001127371.2:c.1119C>G<br>NM_018719.4:c.1257C>G	NM_001127370.2:p.Asp385Glu<br>NM_001127371.2:p.Asp373Glu<br>NM_018719.4:p.Asp419Glu
FADS6	in-frame	0,22	Chr17(GRCh37):g.72889676_72889677ins54	NM_178128.3:c.17_18ins54	NM_178128.3:p.Pro15_Ala16insThrGluProMetGluProThrGluProMetGluProThrGluProMetGluPro
GCN1	missense	0,33	Chr12(GRCh37):g.120578680A>T	NM_006836.1:c.5977T>A	NM_006836.1:p.Ser1993Thr

Supplementary table 3 – In silico pathogenicity predictions and GnomaAD frequency of the *YIPF5* homozygous variants. The in silico tools were accessed through AlamutVisual (species considered: Pan troglodytes, Rattus norvegicus, Mus musculus, Canis familiaris, Ornythorhyncus anatinus, Gallus gallus, Xenopus tropicalis, Tetraodon nigroviridis, Danio rerio, Drosophila melanogaster, Caenorhabditis elegans, Saccharomyces cerevisiae).

Family	Mutation			In silico prediction				Gnomad All Frequency
	gDNA (GRCh37)	cDNA	Protein	SIFT	PolyPhen-2	AlignGVGD	MutationTester	
I	Chr5:g.143541851G>A	c.542C>T	p.(Ala181Val)	Deleterious (0.01)	Benign (0.062)	C0	Disease Causing (1)	0
II	Chr5:g.143543785_143543787del	c.317_319del	p.(Lys106del)	N/A	N/A	N/A	N/A	0
III	Chr5:g.143543811A>C	c.293T>G	p.(Ile98Ser)	Deleterious (0)	Probably Damaging (0.97)	C65	Disease Causing (1)	0
IV	Chr5:123540083TA>T	c.652T>A	p.(Trp218Arg)	Deleterious (0)	Probably Damaging (1)	C65	Disease Causing (1)	0
V	Chr5:143543814C>A	c.290G>T	p.(Gly97Val)	Deleterious (0)	Probably Damaging (1)	C65	Disease Causing (1)	0

Supplementary table 4 – PCR Primers used for variant confirmation and family testing (NM_001024947.3, exon 1 is non-coding)

	FW	RV
*YIPF5*_Exon 2	TTACTGGGCCGCAACTTAAAAGA	GATATGTGACACTGGCTTGGCTT
*YIPF5*_Exon 3	AAATGCTTAGTGCTATGTAAGTGAA	TGTGGTTTCGCATATACTCTGTT
*YIPF5*_Exon 4	AGAGAGCTCACTGAAATAATATCATCC	TGTTCTACCAAAAGCCATTTTGA
*YIPF5*_Exon 5	AGCCAGTCTGCTCAGTCTTTTAC	CTGTCCCTACTACAAGAGGCACT
*YIPF5*_Exon 6	AGCTGTTTATCCCATGAAATGATTC	ACACGTTTACTTTCATTGCTCCAA

Supplementary table 5 – Primers used for hESCs and iPSCs genome-editing.

	FW	RV
**hESCs exon 3 deletion colonies screen primers**	TTTGTTGACCTCCTAACCTTTAAC	AAAGTCGATGCTGACATAGTAACA
**hESCs *YIPF5* p.Ile98Ser mutation template primers**	ATGTCATTTTGTCTCTTGTTTCAGGATAGATTTTGATGA ATTAAAAATACATCACAAATCCTAATTAACTTTTAAATT TTCTTTCTCTTTAGAGCTGGGATCCAACTTCG	TTGGACCTGCCAAATCAGTTTCATTCATGATGCTGCCAT CTGCTACTTTTAACGGATGTAATACTGTTAGTGTTTTCT GCCAGATATGATCGAAGTTGGATCCCAGCTCT
**iPSCs *YIPF5* p.Ile98Ser correction template primers**	ATGTCATTTTGTCTCTTGTTTCAGGATAGATTTTGATGA ATTAAAAATACATCACAAATCCTAATTAACTTTTAAATT TTCTTTCTCTTTAGAGCTGGGaAtCAACTTcG	TTGGACCTGCCAAATCAGTTTCATTCATGATGCTGCCAT CTGCTACTTTTAACGGATGTAATACTGTTAGTGTTTTcTG CCAGATGTGaTCgAAGTTGaTtCCCAGCTCT
***YIPF5* p.Ile98Ser mutation screening in hESCs and iPSCs clones**	ACTGGCATAGAGAGCTCACTGAAA	AGACTTGAGGTGTAAAGCGGAGT
**KO G1 Off-target 1**	AAGAGTAGACCCCTGTGGCA	AGGACGAGTGAAATCAGATGCA
**KO G1 Off-target 2**	TCTTCCCAAGGTTAATGGTAAGC	AATGAGGGGCTGCGTGTTTA
**KO G1 Off-target 3**	CCCACTAATGCTACCATGGCA	GAGACCTGACCCCTCCTTCT
**KO G2 Off-target 1**	ACAACTGGGTAGTATGAAGCCA	TGCCAGAGAGACTTAGGTGAGT
**KO G2 Off-target 2**	GTCTATTGCCCCAAGCCACT	TTCAGGCAGGTAACGTGTCA
**KO G2 Off-target 3**	TGCTGTTTGGGTGTCCTAAGT	AGCAGTGTCCCCTACTGAGT
***YIPF5* Corr Off-target 1**	GCAAGTTAAGTTGTGGACCCA	ACCAGAAGTCAAAGACCAGCA
***YIPF5* Corr Off-target 2**	GCATGTTGCCCCAAATCATCA	CACCAAGCAACTGGCCAATC
***YIPF5* Corr Off-target 3**	CCATGCGTTCTACTAAGGATAGC	AGCTCTATTGGCTGCAGTCT
***YIPF5* Mut Off-target 1**	TTCAAAGGTGCACCCCTTGT	CCCTCTCCCCTCTGCTGTAT
***YIPF5* Mut Off-target 2**	GGCAAAACCAGGCTGTAAGC	AGTAACCCCGATGCTTTCCT
***YIPF5* Mut Off-target 3**	GCTTCACGCAAAACAGGAGG	GTGTTTAGACGGCCACCAGG

Supplementary table 6 – Antibodies used for Fluorescence-activated cell sorting (FACS) for flow cytometry.

Mouse Anti-CD184 (CXCR4) Monoclonal Antibody, Phycoerythrin Conjugated,Clone 12G5	BD Biosciences Cat# 555974; RRID:AB_396267	FC; (1:80)
Mouse IgG2a, kappa Isotype Control, Phycoerythrin Conjugated, Clone G155-178	BD Biosciences Cat# 563023	FC; (1:80)
Insulin (C27C9) Rabbit Antibody (Alexa Fluor 647 Conjugate)	Cell Signaling Technology Cat# 9008; RRID:AB_2687822	FC; (1:80)
Rabbit IgG Isotype Control (Alexa Fluor 647 Conjugate)	Cell Signaling Technology Cat# 3452S; RRID:AB_10695811	FC; (1:80)
Mouse anti-GCG unconjugated antibody	Sigma-Aldrich Cat# G2654; RRID:AB_259852	FC; (1:160)

Supplementary table 7 – qRT PCR primers used in hESC cell-lines.

PPIG	NM_004792	Fw: TCTTGTCAATGGCCAACAGAG;<br>Rv: GCCCATCTAAATGAGGAGTTG; 84 bp
PDX1	NM_000209.3	Fw: AAGTCTACCAAAGCTCACGCG;<br>Rv: CGTAGGCGCCGCCTGC; 52 bp
NKX6.1	NM_006168	Fw: TATTCGTTGGGGATGACAGAG;<br>Rv: TGGCCATCTCGGCAGCGTG; 91 bp
INS	NM_000207	Fw: CAGAAGCGTGGCATTGTGGA;<br>Rv: GCTGCGTCTAGTTGCAGTAG; 82 bp
GCG	NM_002054	Fw: GAAGGCGAGATTTCCCAGAAG;<br>Rv: CCTGGCGGCAAGATTATCAAG; 113 bp
BiP (HSPA5)	NM_005347.4	Fw: TGGCTGGAAAGCCACCAAGATGCT;<br>Rv: GGGGGAGGGCCTGCACTTCCAT; 116 bp
XBP1s	NM_001079539.1	Fw: CTGCTGAGTCCGCAGCAGGTGCA;<br>Rv: GGTCCAAGTTGTCCAGAATGC; 129 bp
CHOP (DDIT3)	NM_001195053.1	Fw: GCACCTCCCAGAGCCCTCACTC;<br>Rv: CCCGGGCTGGGGAATGACCA; 120 bp
ATF6	NM_007348.4	Fw: ACCTGCTGTTACCAGCTACCACCCA;<br>Rv: GCATCATCACTTCGTAGTCCTGCCC; 120 bp
GRP170 (HYOU1)	NM_001130991.2	Fw: GTCCAAGGGCATCAAGGCTC;<br>Rv: TTCTGCGCTGTCCTCTACCA; 103 bp
YIPF5 Exon 3	NM_030799.9	Fw: ATGGAGGAAGTGGAGGACCCTAT;<br>Rv: TGCCCGGTGTATGGCTGTTG; 112 bp

Supplementary table 8 – qRT PCR primers used in EndoC-βH1 cell-line and *YIPF5* patient β-cell-related experiments.

GAPDH	NM_001357943.2	Fw: CAGCCTCAAGATCATCAGCA;<br>Rv: TGTGGTCATGAGTCCTTCCA; 106 bp
ACTB	NM_001101.5	Fw: CTGTACGCCAACACAGTGCT;<br>Rv: GCTCAGGAGGAGCAATGATC; 127 bp
OAZ1	NM_001301020.1	Fw: TACAGCAGTGGAGGGAGACC;<br>Rv: GGATCCTCAATAGCCACTGC; 144 bp
PDX1	NM_000209.4	Fw: AAAGCTCACGCGTGGAAA;<br>Rv: GCCGTGAGATGTACTTGTTGA; 145 bp
NKX6.1	NM_006168.2	Fw: GGGCTCGTTTGGCCTATT;<br>Rv: CGTGCTTCTTCCTCCACTT; 90 bp
NKX2.2	NM_002509.4	Fw: GAACCCCTTCTACGACAGCA;<br>Rv: ACCGTGCAGGGAGTACTGAA; 82 bp
NEUROD1	NM_002500.5	Fw CTATCACTGCTCAGGACCTACT;<br>Rv: CCACTCTCGCTGTACGATTT; 69 bp
INS	NM_001185098.2	Fw: CCAGCCGCAGCCTTTGTGA;<br>Rv: CCAGCTCCACCTGCCCCA; 145 bp
GCG	NM_002054.5	Fw: CTGGGAAATCTCGCCTTCCT;<br>Rv: AACATTGCCAAACGTCACGA; 142 bp
NGN3	NM_020999.4	Fw: GACGACGCGAAGCTCACCAA;<br>Rv: TACAAGCTGTGGTCCGCTAT; 98 bp
SOX9	NM_000346.4	Fw: ATCAAGACGGAGCAGCTGAG;<br>Rv: GGCTGTAGTGTGGGAGGTTG; 100 bp
BiP		Qiagen quantitect primer 00096404
CHOP (DDIT3)		Qiagen quantitect primer 0002278
XBP1s	NM_001079539.1	Fw: CCGCAGCAGGTGCAGG;<br>Rv: GAGTCAATACCGCCAGAATCCA; 70 bp
DP5 (HRK)	NM_003806.4	Fw: GAGCCCAGAGCTTGAAAGG;<br>Rv: CCCAGTCCCATTCTGTGTTT; 99 bp
PUMA (BBC3)	NM_001127240.3	Fw: TTGTGCTGGTGCCCGTTCCA;<br>Rv: AGGCTAGTGGTCACGTTTGGCT; 164 bp
HYOU1	NM_001130991.3	Fw: AAGGCTCACTTCAACCTGGA;<br>Rv: TCTCCTCCTCCTCCTGGACA; 202 bp
BIM (BCL2L11)	NM_001204106.2	Fw: TCCTTGCAGCCACCCTGC;<br>Rv: CTTGCGTTTCTCAGTCCGA; 152 bp
YIPF5	NM_001271732.2	Fw: GTAGCAGATGGCAGCATCAT;<br>Rv: TGCCAGCCAGTAGCAATGTG; 91 bp

Supplementary table 9 – Antibodies used for Immunocytochemistry, Immunohistochemistry, and Western Blot.

Rabbit anti-OCT4	Santa Cruz Biotechnology Cat# sc-9081; RRID:AB_2167703	ICC; (1:500)
Mouse anti-TRA1-60	Thermo Fisher Scientific, RRID:AB_2536699	ICC; (1:50)
Mouse anti-SSEA4	Thermo Fisher Scientific, Cat# MA1-021-D488	ICC; (1:200)
Guinea pig anti-INS	Dako Cat# A0564; RRID:AB_10013624	ICC, IHC; (1:500)
Mouse anti-PROINS	DSHB Cat# GS-9A8; RRID:AB_532383	ICC, IHC; (1:300)
Mouse anti-GCG	Sigma-Aldrich Cat# G2654; RRID:AB_259852	ICC, IHC; (1:500)
Rabbit anti-BiP	Cell Signaling Technology Cat# 3177S; RRID:AB_2119845	ICC, IHC; (1:500)
Rabbit anti-GRP170	abcam, Cat#ab134944; RRID:AB_2858190	ICC, IHC; (1:250)
Rabbit anti-Calreticulin	abcam, Cat#ab2907; RRID:AB_303402	ICC, IHC; (1:250)
Rabbit anti-YIPF5	abcam, Cat#ab102838, RRID:AB_10712568	WB (1:500)
Rabbit anti-GAPDH	Trevigen, Cat#2275-PC-100, AB_2107456	WB (1:1000)
Anti-Rabbit IgG	Jackson Immunoresearch, Cat #711-036-152, RRID:AB_2340590	WB (1:5000)

Supplementary Figure 1: (A) Homozygosity mapping for Cases I and II. Graphical representation of the long runs of homozygosity (>3Mb) in Cases I and II detected using SavvyVcfHomozygosity. (B) Photos of individuals I (at age 5 years) and V (at age 7 months).

A

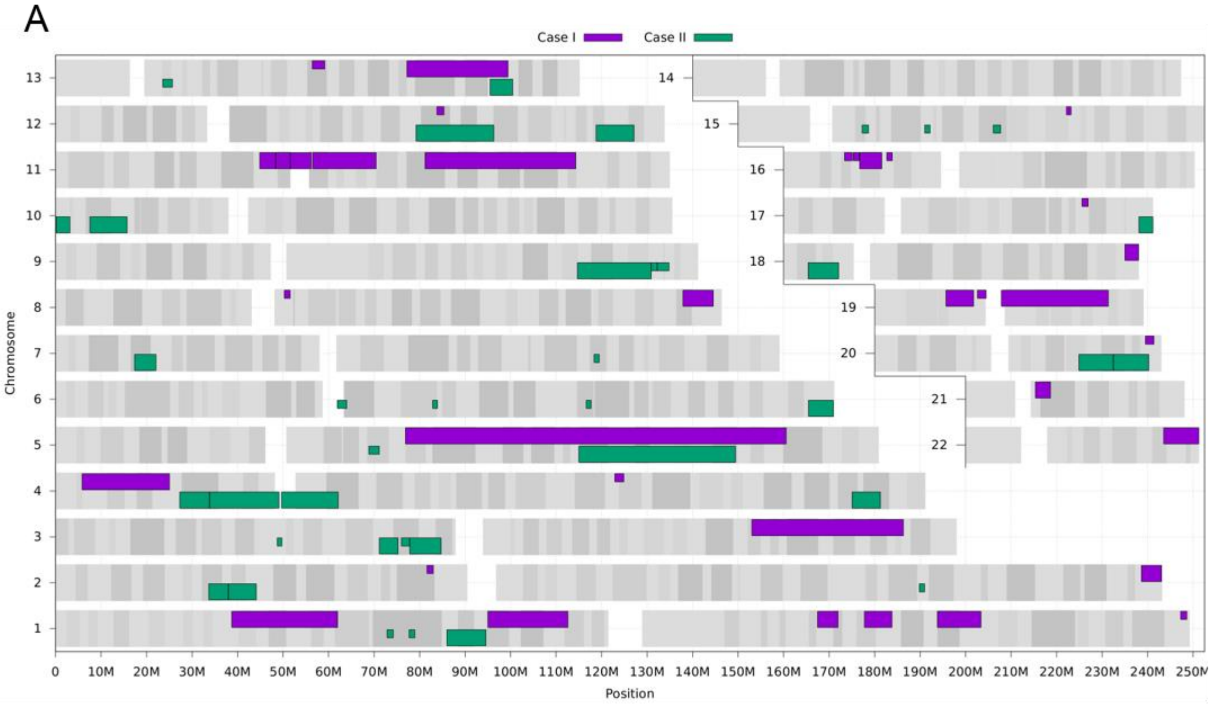

B

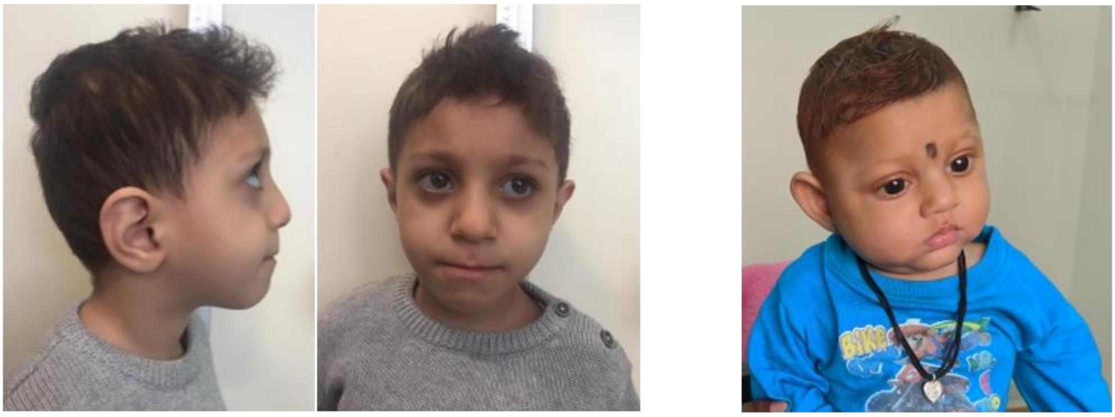

Case I (age 5 years)

Case V (age 7 months)

Supplementary Figure 2: *In vitro* model of *YIPF5*-depleted human EndoC-βH1 cells. (A) *YIPF5* mRNA expression in EndoC-βH1 cells transfected with 2 different siRNAs against *YIPF5* (si1 and si2) or control siRNA (siCT) for 48h and exposed or not (CTL) to the ER stressors thapsigargin (Tha) for 40h or brefeldin A (BFA) for 16h (n=4). (B) YIPF5 mRNA expression in dispersed human islet cells transfected with si1 or siCT for 48h and exposed or not to brefeldin A for 24h (n=4). (C-G) EndoC-βH1 cells were transfected with si1 or siCT for 48h and exposed to thapsigargin for the indicated times (n=5-6). Spliced XBP1 (C), BiP (D), PDIA4 (E), HYOU1 (F) and PUMA (G) mRNA expression as fold change to basal condition. CHOP (H) and DP5 (I) mRNA expression in EndoC-βH1 cells transfected with siCT or si1 and/or siRNA against CHOP (siCHOP) (H) or DP5 (siDP5) (I) and treated or not with thapsigargin for 40h (n=5 and n=8, respectively). mRNA expression was assayed by qPCR and normalized to the reference gene β-actin (ACTB). Paired two-way ANOVA followed by followed by t-tests with the Bonferroni correction for multiple comparisons. *p<0.05, **p<0.01, ***p<0.001 vs siCT in the respective condition. ##p<0.01 and ### p<0.001 for treated vs untreated cells, &&&p<0.001 as indicated.

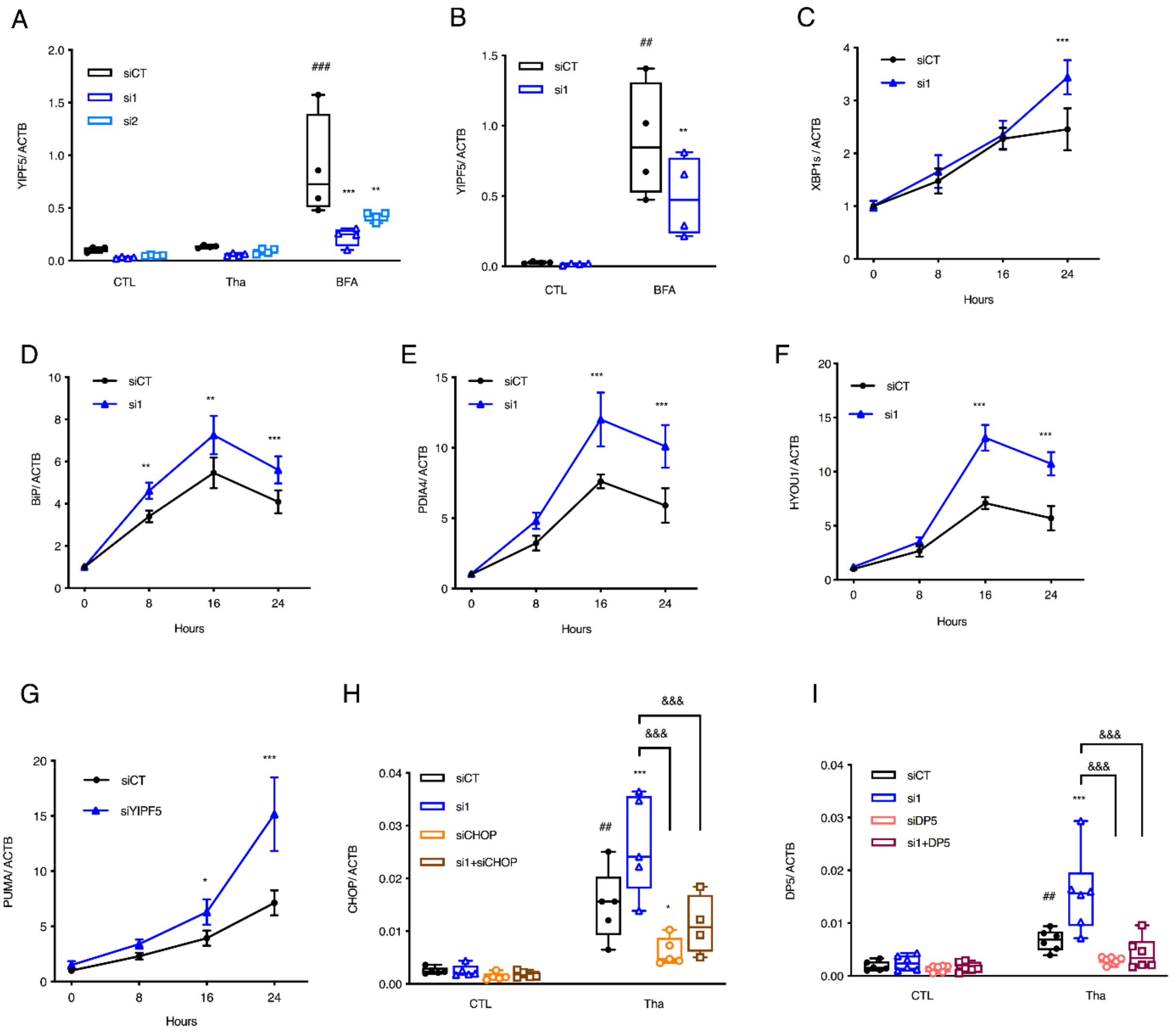

Supplementary Figure 3: YIPF5 knocking-out strategy in human embryonic stem cells.

(A) The third exon was deleted using two CRISPR/Cas9 guides to knockout all YIPF5 isoforms. (B) A 700 bp PCR amplicon of YIPF5 gene showing a 200 bp-deletion in the KO clone.

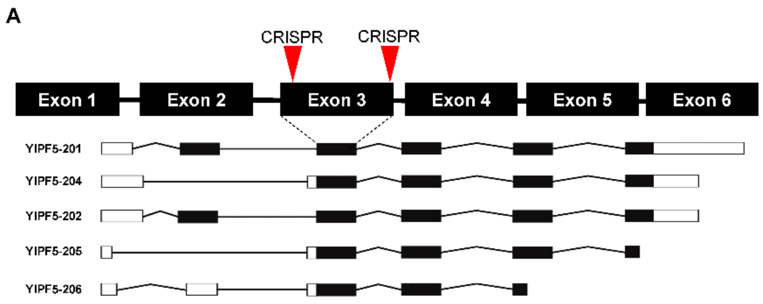

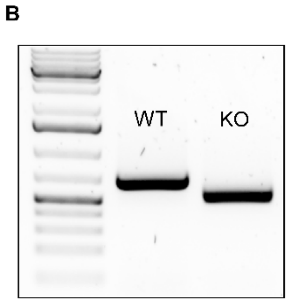

Supplementary Figure 4: Characterization of *YIPF5* KO line post genome editing.

(A) Immunocytochemistry for pluripotency factors OCT4, TRA 1-60 and SSEA4 for both WT and YIPF5 KO cell-lines. (B) Relative mRNA expression levels of pluripotent factors OCT4, SOX2 and NANOG for both WT and YIPF5 KO cell-lines (n=3). Statistical significance was measured using multiple t-test with Bonferroni correction. Error bars represent ±SD from the mean. (C) Normal karyotype visualised with G-banding for the YIPF5 KO clone.

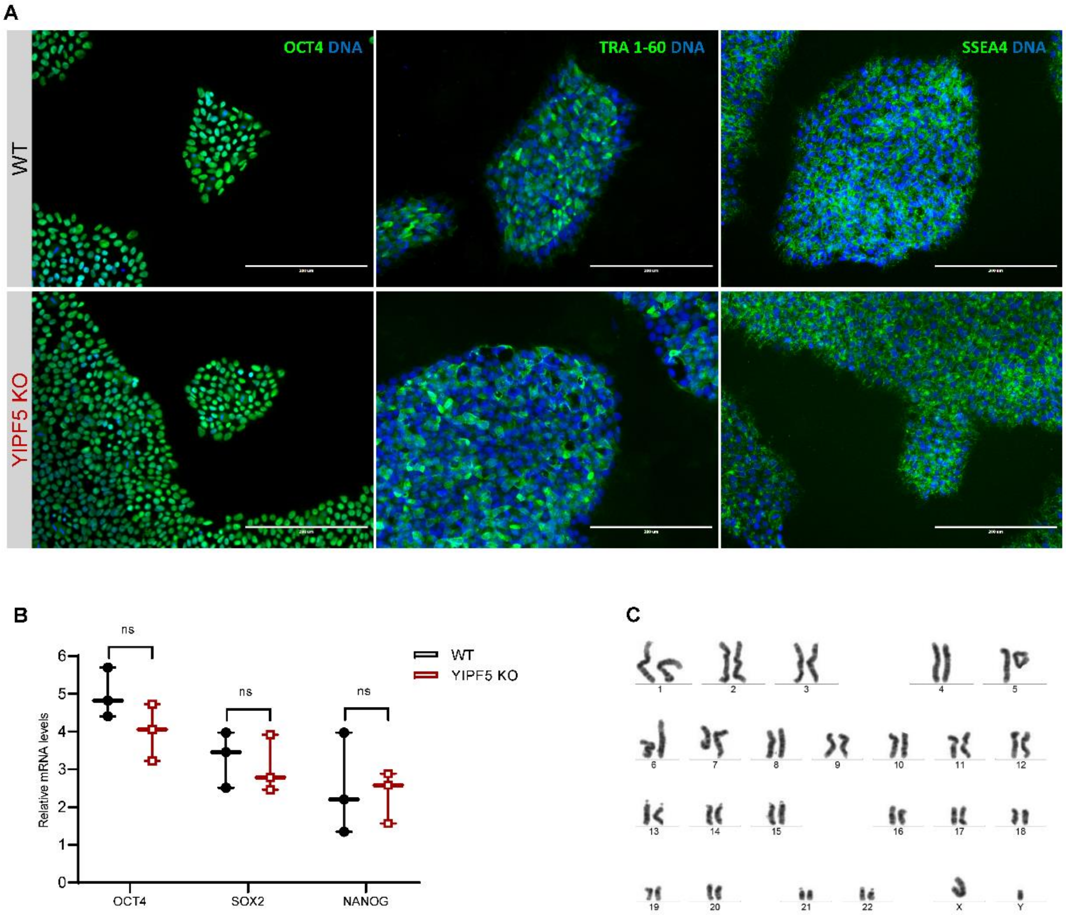

Supplementary Figure 5: Genome editing for the p.(Ile98Ser) mutation in iPSCs and hESCs.

(A) The correction strategy for the patient's mutation p.(Ile98Ser) using CRISPR/Cpf1 stimulated homology directed repair, resulting in a corrected iPSC clone A8. (B) The mutation strategy for introducing p.(Ile98Ser) into the WT H1 cell-line using CRISPR/Cpf1 stimulated homology directed repair, resulting in a corrected iPSC clone G8. (C) Screening of the clones using the generated restriction sites, PfeI for the mutation correction in the iPSC line and BamHI for the mutation insertion in the WT H1 hESCs. (D) Normal iPSCs karyotype visualised with G-banding, (E) immunofluorescent staining of iPSCs for the pluripotency markers OCT4, SSEA4, NANOG and TRA-1-60 and (F) iPSC morphology visualised with bright-field microscopy in the YIPF5 corrected iPSC clones A8 and B9.

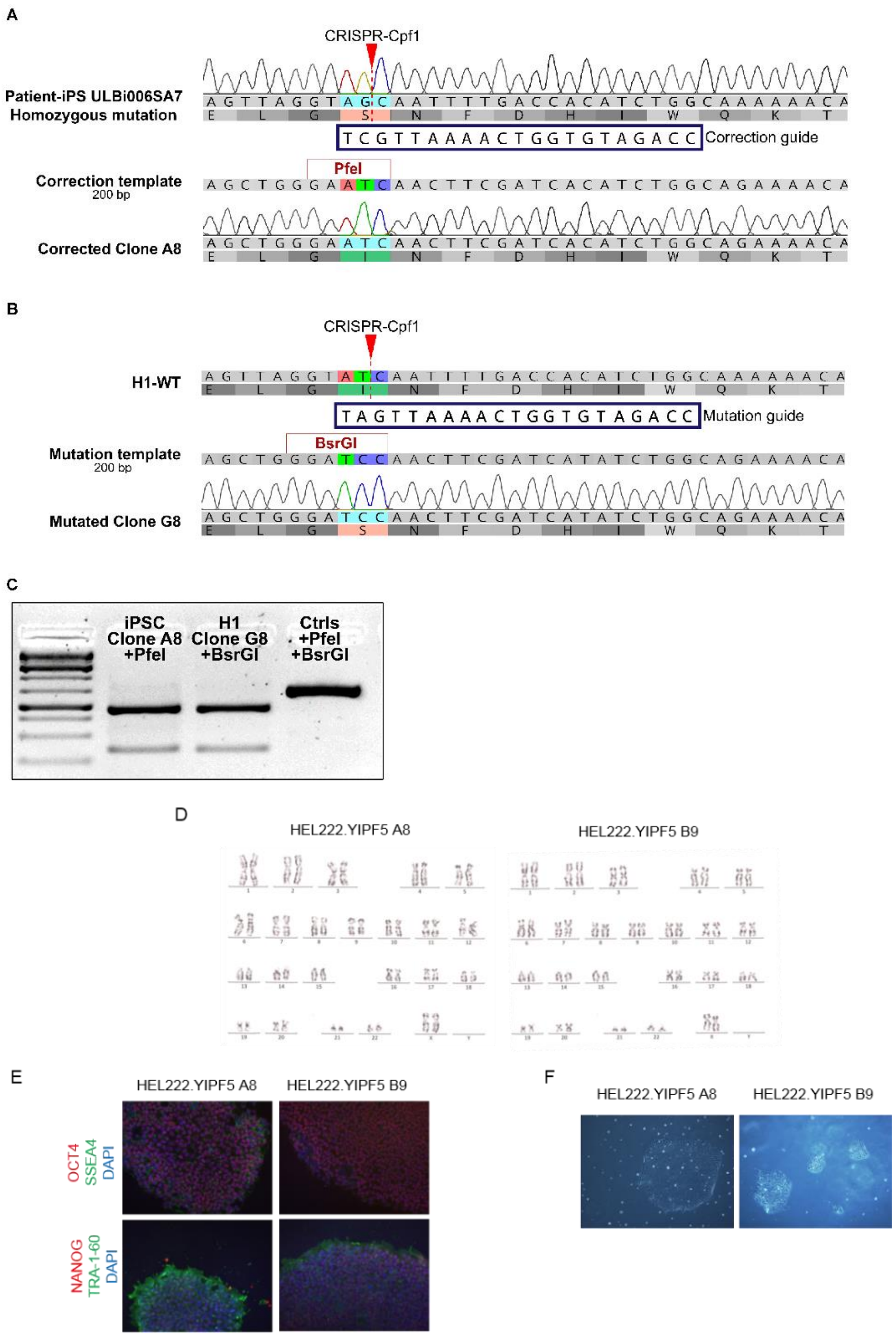

Supplementary Figure 6: Immunocytochemistry showing proinsulin accumulation and severe ER stress in YIPF5 KO β-cells.

(A) Immunocytochemistry for proinsulin (PROINS) and insulin (INS) at stage 7 of in-vitro differentiation for WT, YIPF5 KO and YIPF5^{Ile98Ser} cells. Scale bar = 50 µm. (B) Immunocytochemistry for BiP and insulin (INS) at stage 7 of in-vitro differentiation. Scale bar = 100 µm.

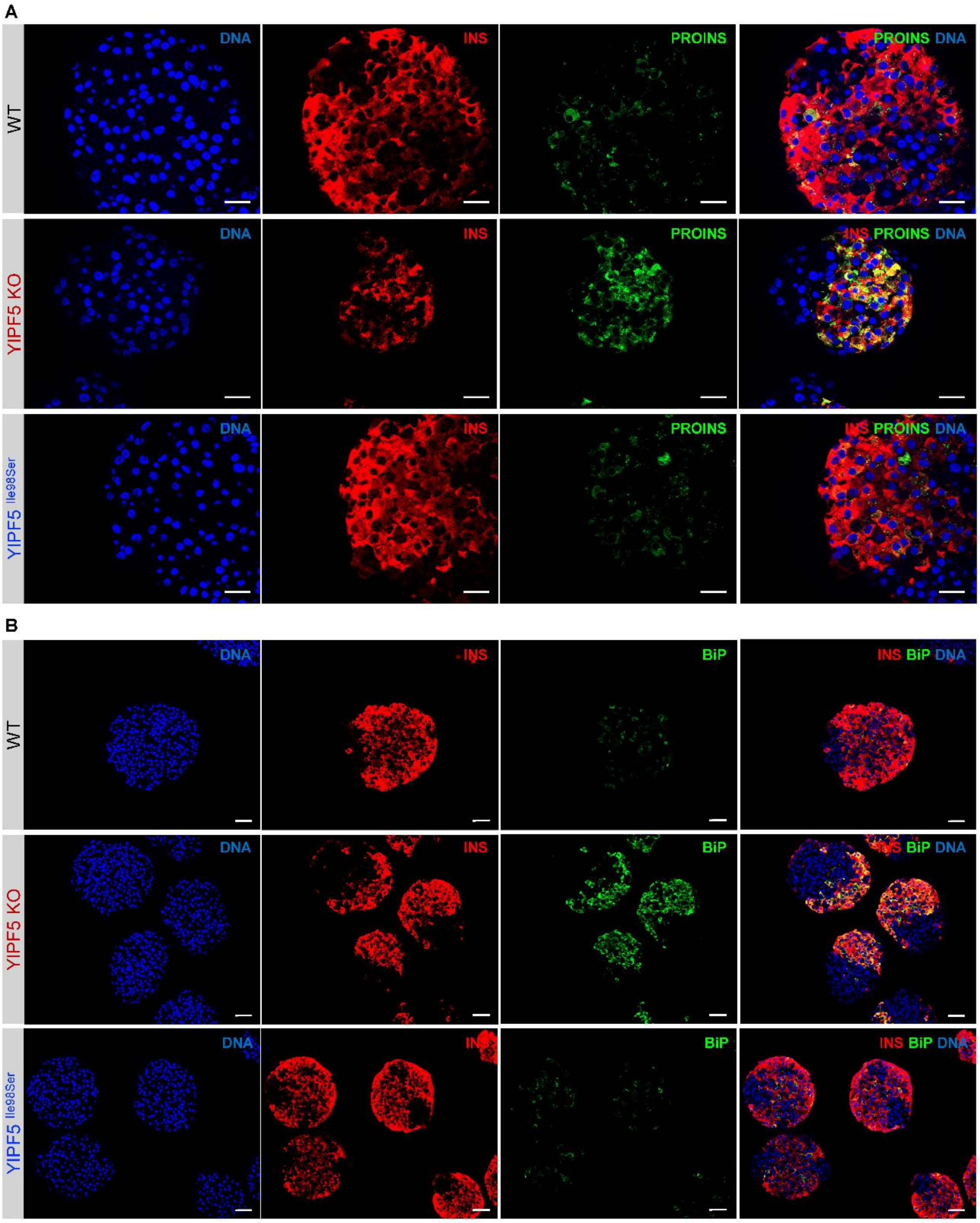

Supplementary Figure 7: mRNA expression fold change during the 7 stages of *in-vitro* differentiation. Pancreatic markers expression values are shown for (*PDX1*, *NKX6.1*, *INS* and *GCG*) and ER-stress markers (*BiP*, *HYOU1*, *XBP1s*, *ATF6* and *CHOP*) (n=3-8). Confirmation of exon 3 deletion on the cDNA level (*YIPF5* Exon 3) (n=1-3). Statistical significance was measured using multiple t-test with Bonferroni correction, *p<0.05, **p<0.01 and ***p<0.001. The mean values are represented with ±SEM error bars.

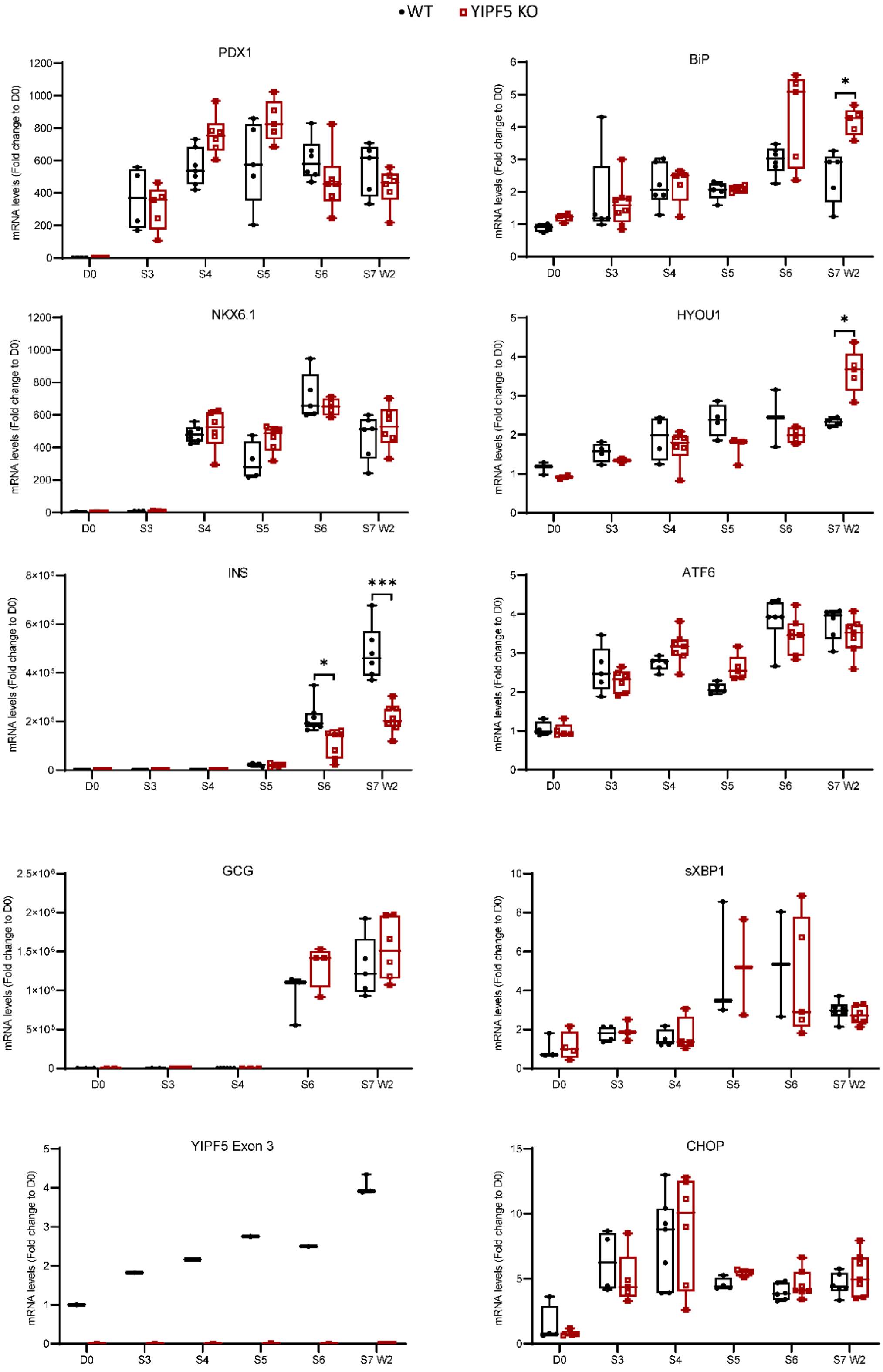

Supplementary Figure 8: Transmission electron microscopy showing severe ER distension in YIPF5 KO β-cells.

(A) Electron micrographs of WT stage 7 cells, where acinar cells are marked with A, β-cells are marked with β and α-cells are marked with α. Yellow arrows point at insulin granules, red arrows point at glucagon granules and green arrows point at the ER. The red box indicates the magnified image in the bottom (B) Electron micrographs of $YIPF5^{Ile98Ser}$ stage 7 cells showing a mild ER distension of β-cells. (C) Electron micrographs of YIPF5 KO stage 7 cells showing severe distension of the ER in β-cells.

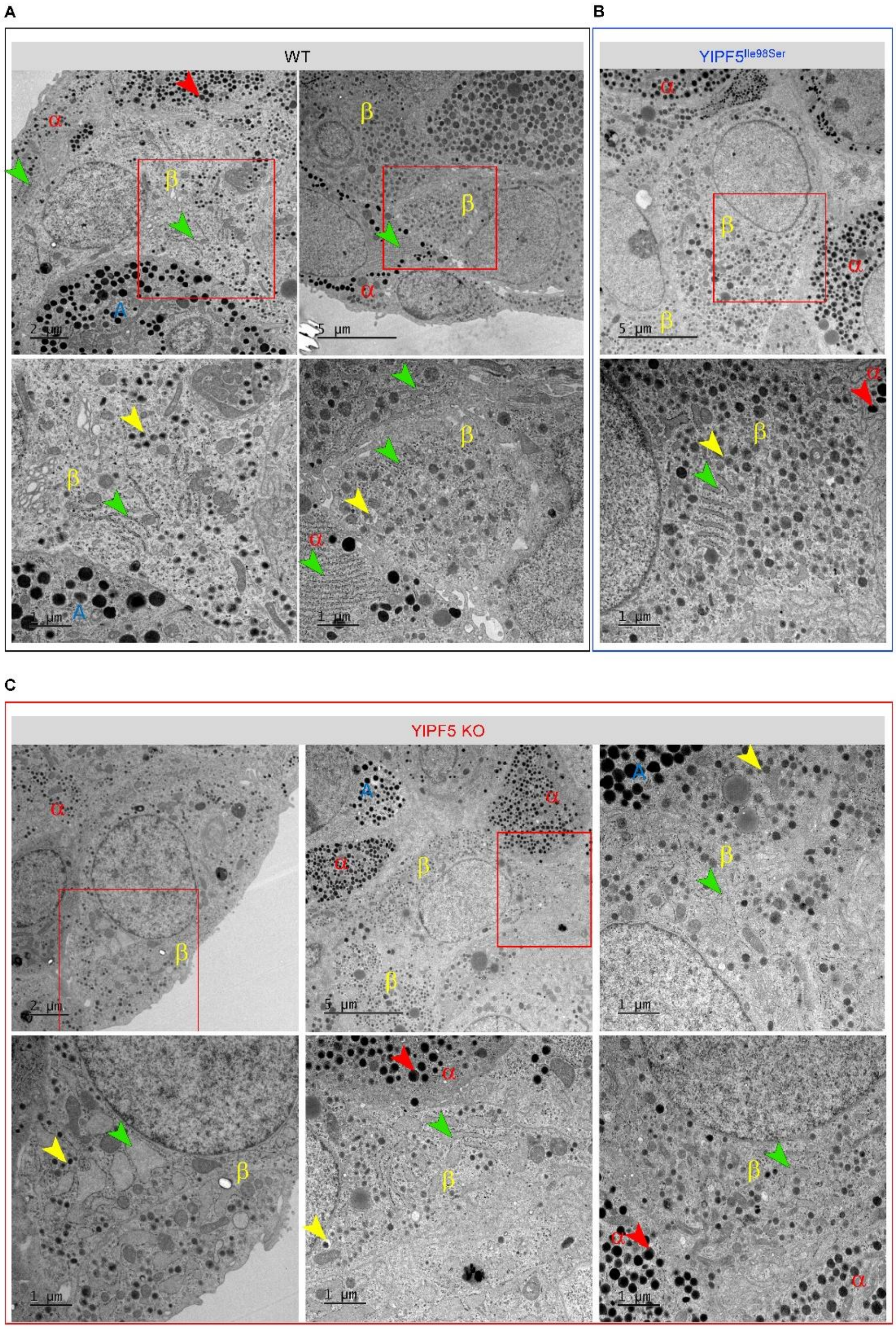

Supplementary Figure 9: Proinsulin accumulation in the ER in YIPF5 KO and YIPF5^{Ile98Ser} β-cells. (A) Immunohistochemistry for calreticulin (CALR) and proinsulin (PROINS) in WT, YIPF5 KO and YIPF5^{Ile98Ser} 3-months grafts. (B) Immunohistochemistry for BiP and proinsulin (PROINS) in 3-months grafts. (C) Immunohistochemistry for GRP170 and proinsulin (PROINS) in 3-months grafts. Scale bar = 25 μm.

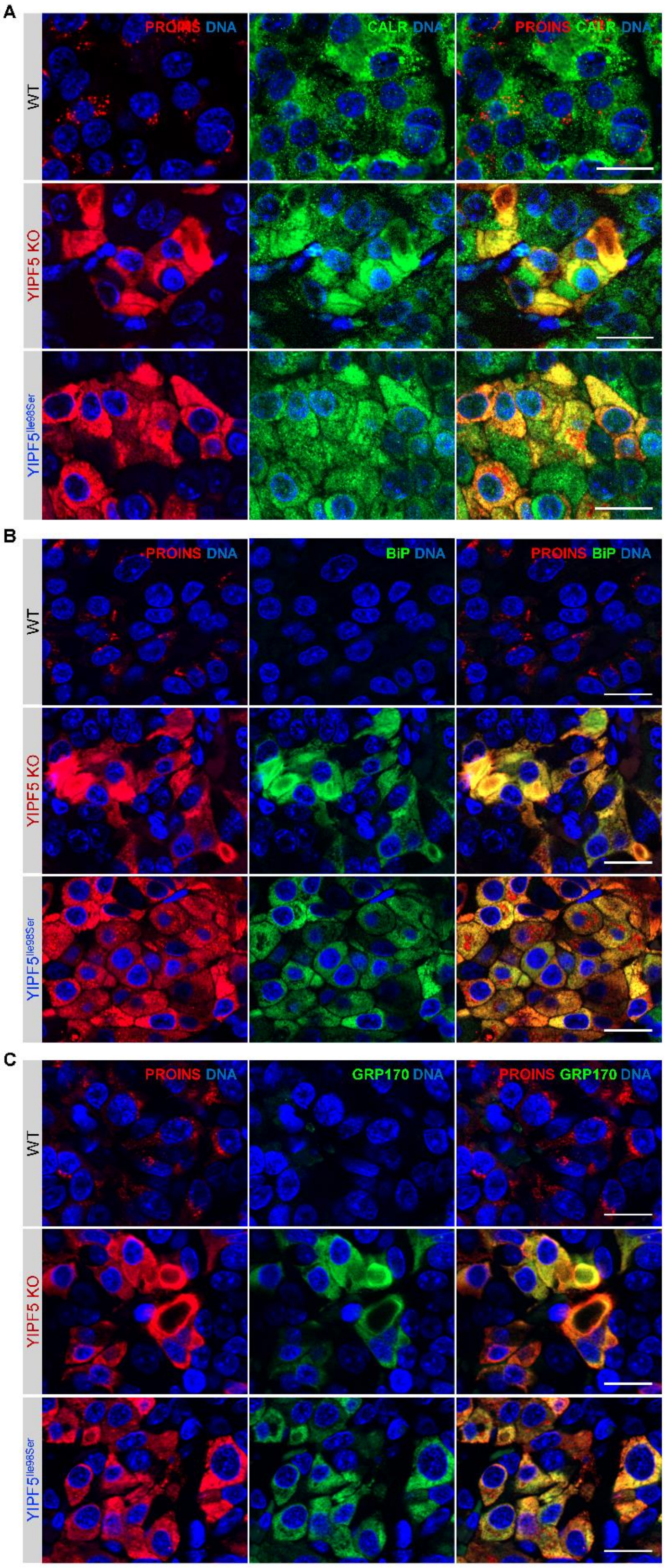

Supplementary Figure 10: Characterization of YIPF5 patient's lines ULBi0006 BA2/BA11/SA2.
(A) Normal iPSC karyotypes visualised with G-banding. (B) Immunofluorescent staining of iPSCs for the pluripotency markers NANOG, OCT4, SSEA4 and TRA-1-60. (C) qPCR showing removal of the transgene vector. cDNA from early passage Sendai vector-transduced PBMCs was used as a positive control. cDNA from ESC line MEL1-hINS was used as negative control. (D) iPSC morphology visualised with bright-field microscopy. (E) Immunofluorescence analysis of embryoid bodies derived from iPSCs. SOX17, vimentin and β-tubulin III were used as markers of endoderm, mesoderm and ectoderm respectively.

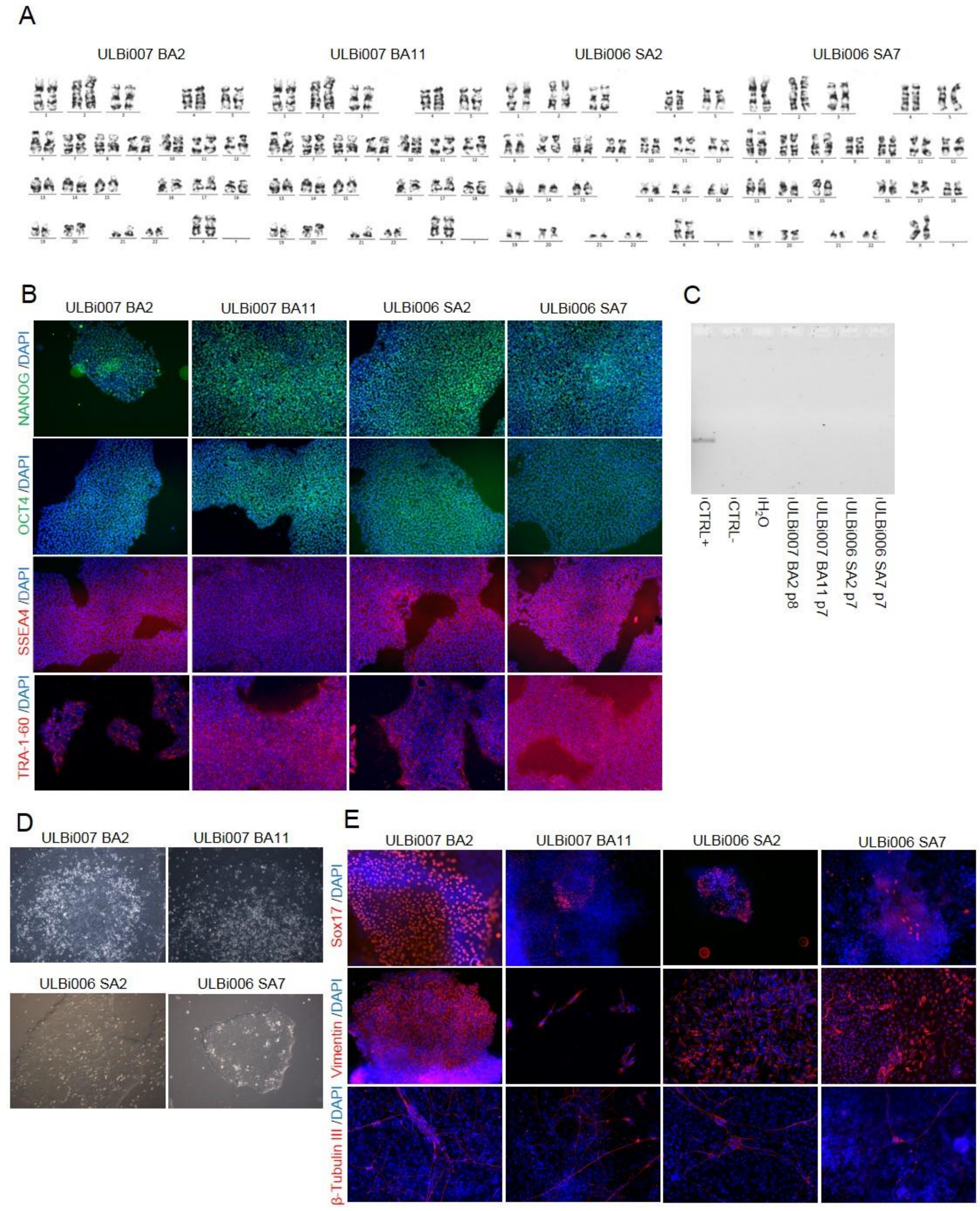

Supplementary Figure 11: Gene expression during iPSC differentiation into pancreatic β-cells. mRNA expression of *YIPF5* patient's lines and control cell lines at iPSC stage (D0) and across differentiation stages 3 to 7 (n=4-12). *SOX9* and *NGN3* are transiently expressed at stage 4 and 5, respectively, followed by induction of downstream genes *NKX2.2* and *NEUROD1*. *PDX1*, *NKX6.1*, *INS* and *GCG* are progressively expressed. Data were normalized to the geometric mean of the reference genes *β-ACTIN* and *GAPDH*. Individual data points represent independent experiments. The median is shown by horizontal lines in the box plots; 25th and 75th percentiles are at the bottom and top of the boxes; whiskers represent minimum and maximum values. Blue squares represent patient cells (2 patients, 2 iPSC lines for each); black circles and squares represent healthy control (1 iPSC line) and corrected patient cells (2 iPSC lines from 1 patient), respectively.

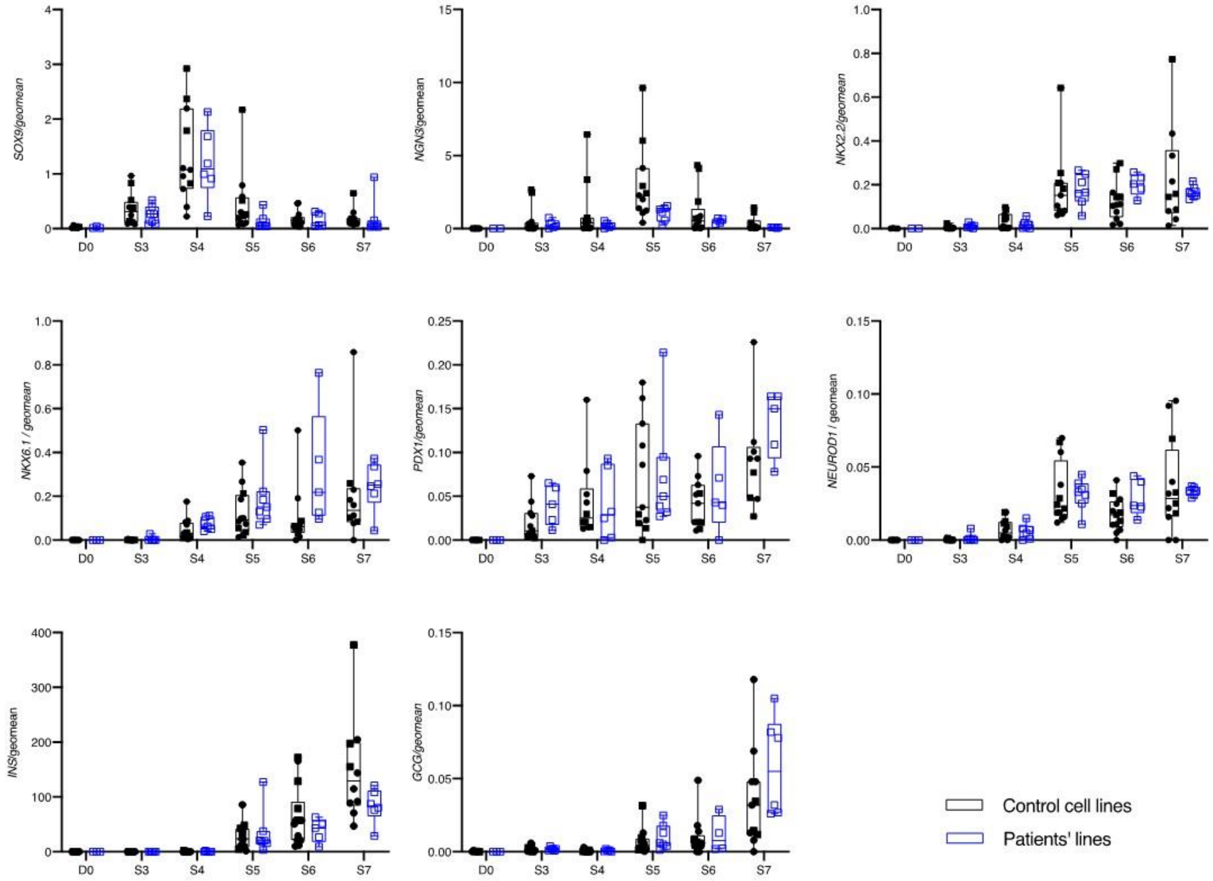

Supplementary Figure 12: Proinsulin and insulin levels in stage 7 aggregates derived from *YIPF5* patients' lines and control lines. (A-B) Quantification of proinsulin (A) and insulin (B) content (pmol) normalized to total protein (mg). N=13-14 and n=6 for control/corrected and patients' lines, respectively. (C) Glucose-stimulated insulin release after exposure to low glucose (1.6 mM), high glucose (16.7 mM) or high glucose plus forskolin (high+Fk, 10 µM). Data were normalized to total protein (mg). N=14 and n=6 for control/corrected and patients' lines, respectively. The median is shown by horizontal lines in the box plots; 25th and 75th percentiles are at the bottom and top of the boxes; whiskers represent minimum and maximum values. Individual data points represent independent experiments. Blue squares represent patient cells (2 patients, 2 iPSC lines for each); black circles and squares represent healthy control (1 iPSC line) and corrected patient cells (2 iPSC lines from 1 patient), respectively.

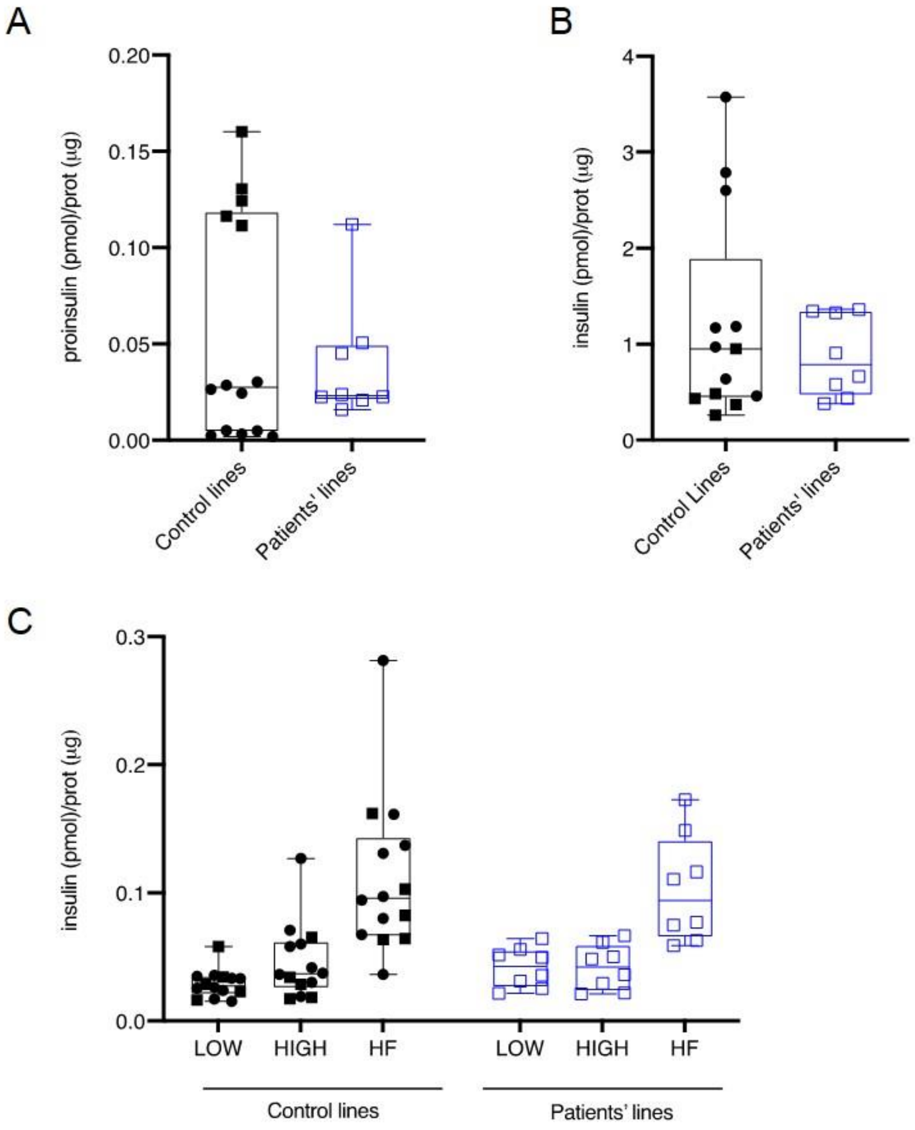

Supplementary References

1. Lambot MA, Depasse F, Noel JC, and Vanderhaeghen P. Mapping labels in the human developing visual system and the evolution of binocular vision. *J Neurosci.* 2005;25(31):7232-7.
2. Saarimaki-Vire J, Balboa D, Russell MA, Saarikettu J, Kinnunen M, Keskitalo S, et al. An Activating STAT3 Mutation Causes Neonatal Diabetes through Premature Induction of Pancreatic Differentiation. *Cell Rep.* 2017;19(2):281-94.
3. Brozzi F, Gerlo S, Grieco FA, Juusola M, Balhuizen A, Lievens S, et al. Ubiquitin D Regulates IRE1alpha/c-Jun N-terminal Kinase (JNK) Protein-dependent Apoptosis in Pancreatic Beta Cells. *J Biol Chem.* 2016;291(23):12040-56.
4. McQuin C, Goodman A, Chernyshev V, Kamentsky L, Cimini BA, Karhohs KW, et al. CellProfiler 3.0: Next-generation image processing for biology. *PLoS Biol.* 2018;16(7):e2005970.
